# Pharmacological Characterization of µ-Opioid Receptor Agonists with Biased G Protein or β-Arrestin Signaling, and Computational Study of Conformational Changes during Receptor Activation

**DOI:** 10.3390/molecules26010013

**Published:** 2020-12-22

**Authors:** Justyna Piekielna-Ciesielska, Roberto Artali, Ammar A. H. Azzam, David G. Lambert, Alicja Kluczyk, Luca Gentilucci, Anna Janecka

**Affiliations:** 1 Department of Biomolecular Chemistry, Medical University of Lodz, Mazowiecka 6/8, 92-215 Lodz, Poland; justyna.piekielna@umed.lodz.pl; 2 Scientia Advice, di Roberto Artali, Desio, 20832 Monza and Brianza, Italy; roberto.artali@scientia-advice.com; 3 Department of Cardiovascular Sciences, University of Leicester, Anaesthesia, Critical Care and Pain Management, Leicester Royal Infirmary, Leicester LE27LX, UK; aaha5@leicester.ac.uk (A.A.H.A.); dgl3@leicester.ac.uk (D.G.L.); 4 College of Pharmacy, University of Babylon, Babylon 51002, Iraq; 5 Faculty of Chemistry, University of Wroclaw, F. Joliot-Curie 14, 50-383 Wroclaw, Poland; alicja.kluczyk@chem.uni.wroc.pl; 6 Department of Chemistry “G. Ciamician”, University of Bologna, Via Selmi 2, 40126 Bologna, Italy

**Keywords:** opioid peptides, biased signaling, G-protein, β-arrestin, molecular modeling

## Abstract

In recent years, G protein vs. β-arrestin biased agonism at opioid receptors has been proposed as an opportunity to produce antinociception with reduced adverse effects. However, at present this approach is highly debated, a reason why more information about biased ligands is required. While the practical relevance of bias in the case of µ-opioid receptors (MOP) still needs to be validated, it remains important to understand the basis of this bias of MOP (and other GPCRs). Recently, we reported two cyclopeptides with high affinity for MOP, the G protein biased Dmt-c[d-Lys-Phe-*p*CF_3_-Phe-Asp]NH_2_ (F-81), and the β-arrestin 2 biased Dmt-c[d-Lys-Phe-Asp]NH_2_ (C-33), as determined by calcium mobilization assay and bioluminescence resonance energy transfer-based assay. The biased character of F-81 and C-33 has been further analyzed in the [^35^S]GTPγS binding assay in human MOP-expressing cells, and the PathHunter enzyme complementation assay, used to measure β-arrestin 2 recruitment. To investigate the structural features of peptide-MOP complexes, we performed conformational analysis by NMR spectroscopy, molecular docking, and molecular dynamics simulation. These studies predicted that the two ligands form alternative complexes with MOP, engaging specific ligand–receptor contacts. This would induce different displays of the cytosolic side of the seven-helices bundle, in particular by stabilizing different angulations of helix 6, that could favor intracellular coupling to either G protein or β-arrestin.

## 1. Introduction

Much effort has been devoted to understanding the molecular mechanism by which opioid agonists such as morphine, oxycodone, and fentanyl, produce analgesia but also several adverse effects, i.e., respiratory depression, constipation, tolerance, dependence, and addiction [1,2,3,4]. These agonists mainly act through the μ-opioid receptor (MOP), a G protein-coupled receptor (GPCR) member of the largest family of proteins in the human proteome. GPCRs are integral membrane proteins which consist of seven-transmembrane helical segments with an extracellular N-terminus and an intracellular C-terminus. Activation of a GPCR by an extracellular ligand leads to conformational changes of the receptor which in turn activate internal signal transduction pathways, causing cellular responses. Receptor interactions are terminated by phosphorylation, followed by recruitment of β-arrestins [5], and receptor desensitization or internalization [6,7]. However, β-arrestins are also capable of transducing signals to multiple effector pathways and stimulating independent cell signaling [8]. It has been proposed that GPCRs may activate distinct biochemical pathways depending on the recruitment of either G proteins or β-arrestin.

Early experiments conducted with β-arrestin 2 knockout animals suggested that MOP activation produced analgesia, but significantly fewer side effects [9]. This observation encouraged the emergence of a school of thought that G protein-induced signaling is required for the analgesic effects of opioids, while β-arrestin recruitment is responsible for the unwanted side effects. Ligands able to stimulate GPCRs to engage selective interactions with these effectors have been identified, and are referred to as “biased agonists” [10,11,12]. Morphine is reputed to be unbiased, which is in line with its clinical profile. The concept of biased opioid ligands attracted a lot of attention as a new strategy for the development of more effective and better tolerated drugs [13,14]. Recent evidence from X-ray analyses supports that, upon binding to a GPCR, an agonist can stabilize diverse conformations of the receptor [15,16].

The first reported G protein-biased ligand at MOP was (*R*)-TRV130 (oliceridine). This compound caused only limited β-arrestin recruitment and receptor internalization [17]. TRV130 seemed to elicit strong antinociception in rodents while not inhibiting gastrointestinal transit nor causing depression of the respiratory system when tested at equi-analgesic doses to morphine [10]. TRV130 was co-administered with fentanyl, an opioid known to produce a significant β-arrestin recruitment, and as a result a significantly stronger antinociception in mice was observed without development of tolerance [18].

**Figure 1:**
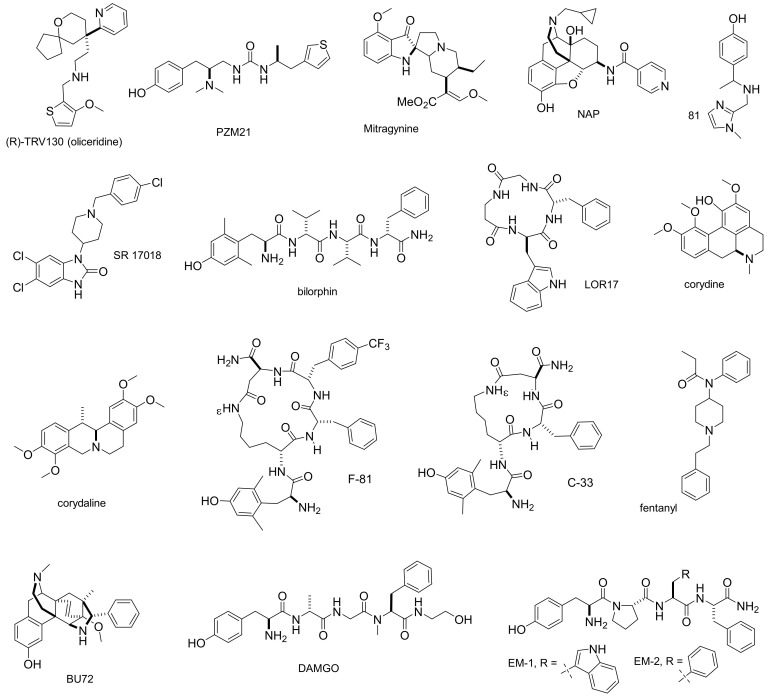
Structures of cyclic peptides F-81 and C-33 and other opioid ligands discussed in this paper.

Following TRV130, diverse biased agonists were identified and characterized (Figure 1). The MOP ligand PZM21 (Figure 1), discovered by in-silico screening [19], was reported to be a potent Gi activator with minimal β-arrestin 2 recruitment. PZM21 was proposed as an analgesic with unprecedented profile and reduced side effects in the hot-plate assay, but not in the tail-flick test. Mitragyna alkaloids (Figure 1) emerged as partial G-protein-biased agonists of the human MOP and competitive antagonists at κ- and δ-opioid receptor (KOP, DOP), which do not recruit β-arrestin 2 following receptor activation [20]. The naltrexamine derivative NAP (NAP, 17-cyclopropylmethyl-3,14β-dihydroxy-4,5α-epoxy-6β-[(4′-pyridyl)acetamido]morphinan, Figure 1) acted at MOP as a low efficacy partial agonist in the G protein-mediated [^35^S]GTPγS binding assay, whereas it did not significantly induce calcium flux or β-arrestin 2 recruitment, and potently blocked MOP full agonist-induced β-arrestin 2 recruitment and translocation [21]. The TRV130 derivative SHR9352 exhibited excellent MOP activity as a G protein-biased agonist, and limited β-arrestin 2 recruitment, as well as a high selectivity over KOP and DOP, and demonstrated robust in vivo efficacy and favorable pharmacokinetic properties [22]. Virtual screening of available compound libraries based on the crystal structure of KOP yielded 81 (Figure 1), a potent Gi biased agonist for KOP with minimal β-arrestin 2 recruitment [23]. Schmid et al. synthesized the opioid SR-17018 (Figure 1) that preferentially engaged G-protein signaling. The authors demonstrated that SR-17018 produced antinociception with minimal respiratory depression relative to fentanyl [24]. SR-17018 showed the premises for the treatment of opioid dependence while restoring opioid antinociceptive sensitivity [25]. Bilorphin (Figure 1), a synthetic tetrapeptide extracted from an Australian fungus, is a potent and selective G protein-biased MOP agonist, marginally recruiting β-arrestin, with no receptor internalization. A glycosylated analog of bilorphin was orally active, with similar in vivo potency to morphine but improved safety [26]. Very recently, the cyclotetrapeptide LOR17, c[Phe-Gly-β-Ala-d-Trp] (Figure 1), lacking a protonable amino group [27], emerged as a novel KOP-selective partial agonist displaying functional selectivity toward G protein signaling and eliciting antinociceptive/antihypersensitivity effects in different animal models, without altering motor coordination, locomotor, and exploratory activities or inducing pro-depressant-like behavior [28]. Finally, the naturally occurring alkaloids corydine and corydaline were identified by virtual screening protocols (Figure 1). Experimentally, these compounds acted as G protein-biased agonists to the MOP without inducing β-arrestin 2 recruitment upon receptor activation, and produced antinociception in mice after subcutaneous administration [29].

Despite the extremely promising preclinical in vivo data, TRV130 had underwhelming results in clinical trials [30], demonstrating only a trend toward reduced side effects, which were not significantly different from morphine. In spite of its alleged bias, the safety profile of oliceridine is similar to other opioids; the most common side effects are nausea, vomiting, dizziness, headache and constipation. Eventually, TRV130 was approved for intravenous use in moderate to severe pain in adults [31]. Contradictory results were reported also for PZM21 by Hill et al. [32]. These authors classified PZM21 as a low efficacy MOP agonist for both G protein and β-arrestin 2 signaling, which depressed respiration and induced tolerance to antinociception in a manner similar to morphine.

In a recent study, a non-phosphorylatable version of MOP was knocked in into mice [33], showing that total abolishment of β-arrestin binding improved analgesia and diminished tolerance, but worsened opioid side effects. These new data supported that respiratory depression and constipation are driven by G protein-mediated signaling, not by β-arrestin-mediated signaling, as was previously assumed based on indirect evidence [9]. This might explain why presumably G protein-biased TRV-130 and PZM21 have a safety profile similar to other unbiased opioids.

These failures led to much debate about the promise of biased agonism at MOP and the numerous reports of new biased agonists in the literature [34]. Some researchers consider that the distinction between desired analgesic effects and unwanted side effects may not be as simple as G protein-mediated vs. β-arrestin-mediated effects [35,36]. As a matter of fact, many compounds characterized by good therapeutic indexes are indeed MOP partial agonists, whose efficacy is likely attributable to low efficacy partial agonism rather than G protein-bias [20,21,27,37,38,39].

In search for alternative pharmacokinetic and pharmacodynamic explanations, it was proposed that kinetics could be important for the observed bias in GPCRs [40]. This opinion mainly stems from studies on the dopamine D2 receptor, a member of the GPCR class, showing that agonist-dependent signaling bias can change dramatically with time, and that this characteristic was linked to the binding kinetics of the agonists [41,42]. As for MOP, very recently Pedersen et al. [43] analyzed the two major pathways, i.e., the activation of Gαi2 and GαoA and the recruitment of β-arrestin 1 and β-arrestin 2 [5], of the biased agonists (*R*)-TRV130, (*S*)-TRV130, buprenorphine, morphine, loperamide, and DAMGO (H-Tyr-d-Ala-Gly-N(Me)Phe-Gly-OH, Figure 1). While all agonists but (*S*)-TRV130 potently activated both G proteins, the latter was 90-fold less potent compared to its (*R*)-isomer. DAMGO recruited both β-arrestin 1 and β-arrestin 2 as a full agonist, whereas morphine and loperamide acted as partial agonists. Buprenorphine and the TRV130 enantiomers failed to recruit any of the β-arrestins to a significant extent, hence showing a remarkable bias for G protein. The authors demonstrated that the bias profile was not time-dependent and that agonists displayed the same degree of bias in spite of strong differences in their binding kinetics. As a matter of fact, the analyses of binding kinetics revealed that DAMGO, morphine, loperamide, (*R*)-TRV130, and (*S*)-TRV130 had dissociation rates from the receptors of the same magnitude, while buprenorphine dissociated much more slowly than the other ligands, with a residence time 18-fold higher than that of TRV130. In summary, considering the differences in dissociation rates in relation to their G protein biased nature, it was concluded that bias was driven by different conformational states of the receptor, rather than by the *k*_off_ values.

Although our understanding of biased ligand utility may be revaluated in the future, there is presently great interest in the investigation of the action mechanisms of biased ligands at the opioid receptors [37]. In this scenario, we studied the interactions of MOP with G-protein and β-arrestin 2, by means of the bioluminescence resonance energy transfer (BRET) assay, which measures the energy transfer between Renilla luciferase (RLuc) linked to the MOP and Renilla green fluorescent protein (RGFP) linked to the signal transducer proteins (G-protein or β-arrestin 2). We identified two cyclopeptides, Dmt-c[d-Lys-Phe-*p*-CF_3_-Phe-Asp]NH_2_ (F-81) [44], which selectively activated G protein, and Dmt-c[d-Lys-Phe-Asp]NH_2_ (C-33) (Figure 1) [45], which displayed a significant bias toward β-arrestin 2.

In this paper, these cyclopeptides have been fully characterized by HPLC, HRMS, ^1^H-NMR, and ^13^C-NMR spectroscopy, and their biased profile has been further analyzed by the [^35^S]GTPγS binding assay and the PathHunter enzyme complementation assay. To investigate possible ligand-receptor interactions responsible for biased signaling, in this study we present the conformational analysis of F-81 and C-33 by NMR and molecular dynamics, and by molecular docking and molecular dynamics of peptide-MOP complexes.

## 2. Results

### 2.1. Pharmacological Investigations

The affinity of F-81 and C-33 for the opioid receptors, and their functional activity determined by the calcium mobilization assay in cells expressing human MOP and chimeric G proteins, previously reported in separate papers [44,45], are resumed for easy comparison in the Supporting Information (Tables S2 and S3). In brief, F-81 showed nanomolar MOP affinity (Ki = 2.4 nM), but also affinity for KOP binding sites (Ki = 31.1 nM), while C-33 showed sub-nanomolar affinity for MOP (Ki = 0.24 nM) and nanomolar affinity for DOP (Ki = 1.33 nM). In the calcium mobilization assay, compounds F-81 and C-33 showed higher potency than the reference EM-2 (Figure 1, Tyr-Pro-Phe-Phe-NH_2_) (2- and 5-fold, respectively). The biased agonism was preliminarily investigated by BRET assay. F-81 appeared to be strongly biased toward G protein and did not promote receptor/β-arrestin interaction. C-33 behaved as a full agonist and promoted MOP/G protein interaction with 8-fold higher potency than EM-2, but displayed, also, a 14-fold bias toward β-arrestin 2 (Table S4).

**Figure 2:**
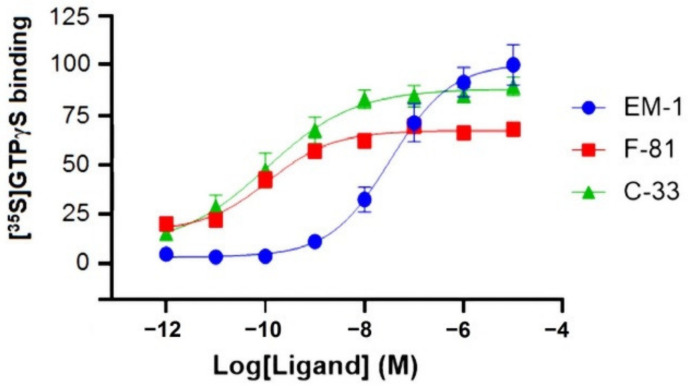
[^35^S]GTPγS functional assay in CHO-hMOP cell membranes. Stimulation of [^35^S]-GTPγS binding to the human MOP by EM-1, C-33, and F-81. Data are normalized to the maximum stimulation caused by EM-2 (100%); values are expressed as mean ± SEM, *n* = 5.

Herein, for the first time the biased agonism of F-81 and C-33 is analyzed by [^35^S]GTPγS binding assay (Table 1) and PathHunter enzyme complementation assay (Table 2). [^35^S]GTPγS binding assay, which characterizes ligand-driven G protein activation, was performed in CHO cells stably expressing the human MOP [46,47]. The stimulatory effects of F-81 and C-33 were compared to EM-1 (Figure 1) used as control; usually, the endogenous agonists produce efficient activation of G protein and β-arrestin 2, and are therefore assumed to be non-biased [48]. Potencies (pEC_50_) and efficacies (% normalized to the maximum stimulation caused by EM-1) are shown in Table 1 and Figure 2. The tested peptides produced a concentration-dependent increase in the [^35^S]GTPγS binding with high potencies, in the sub-nanomolar range. Compound C-33 showed slightly reduced efficacy compared to that of EM-1, whereas F-81 behaved as a partial agonist, with an efficacy of 68% compared to EM-2.

To determine β-arrestin recruitment, F-81 and C-33 were evaluated for their potential stimulatory effects using the PathHunter enzyme complementation assay, with EM-1 as a reference [38,49]. Since in most studies reported in the introduction, β-arrestin 2 was used [19,21,22,23,29], for easy comparison we decided to characterize this isoform, despite being the minority as compared to β-arrestin 1 in most cells, including neurons. The use of β-arrestin 2 is supported by its similarity in opioid receptor binding with β-arrestin 1 [37,50]. Very recently, Pedersen et al. measured the recruitment of both arrestins 1 and 2 following the activation of MOP by various ligands, and similar results were obtained [43].

**Figure 3:**
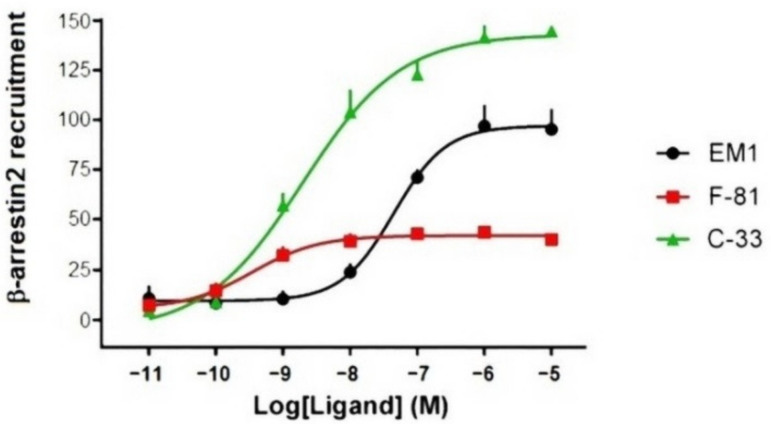
Ligand induced β-arrestin 2 recruitment in CHO-K1 OPRM1 β-arrestin 2 expressing cells by EM-1, C-33, and F-81. Data are normalized to the maximum stimulation caused by EM-1 (100%); values are expressed as mean ± SEM, n = 5.

The tested compounds stimulated the β-arrestin 2 signaling pathway in a concentration-dependent manner (Figure 3 and Table 2). Compared to EM-1 (Tyr-Pro-Trp-Phe-NH_2_), C-33 behaved as a full agonist, with an efficacy of 156%, while and F-81 was the least efficacious, 41% as compared to EM-1. Significant differences in pEC_50_ values were observed for all compounds.

### 2.2. Conformational Analysis

**Figure 4:**
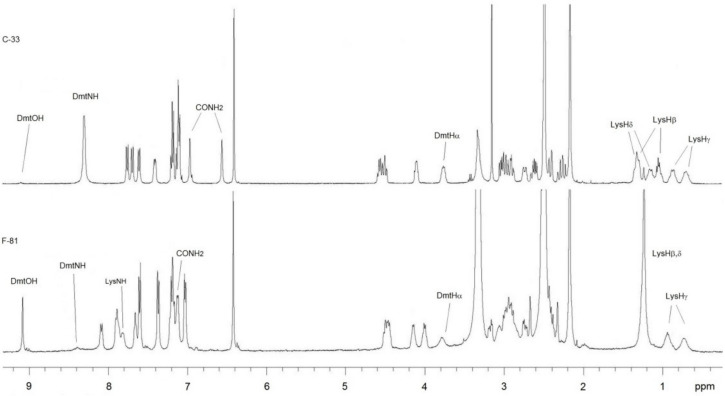
^1^H-NMR spectra at 400 MHz in 8:2 DMSO-*d*_6_/H_2_O of C-33 and F-81; peaks flattened by water pre-saturation are also indicated.

The in-solution structures of the cyclopeptides were analyzed by NMR spectroscopy in 8:2 DMSO-*d*_6_/H_2_O (Figure 4), a solvent mixture generally utilized as a good physical approximation of the mechanical and electrostatic environment at the opioid receptors [51]. All resonances were attributed by 2D gCOSY. The ^1^H-NMR of C-33 showed a single set of sharp resonances, indicating conformational homogeneity or a rapid equilibrium between conformers.

In particular, the resonances of LysHβ,γ,δ appeared well distinct and separated. In contrast, the ^1^H-NMR of F-81 revealed broad peaks for the residues Lys and Dmt, and superimposed LysHβ,δ signals, suggestive of higher flexibility of the Lys linker.

To detect if amide protons take part in intramolecular hydrogen-bonding or are solvent exposed, VT-NMR analysis was performed for both analogs (Table 3). In DMSO-*d*_6_, C-33 showed a comparatively lower Δδ/Δt = −1.6 ppb/K for the CONH_2_ at 6.60 ppm, with respect to the other amide protons, suggestive of a preference for conformations having this CONH_2_ involved in a hydrogen bond (|Δδ/Δt| < or close to 2.0 ppb/K). As for F-81, the chemical shift of PheNH was significantly less sensitive to increasing temperature, Δδ/Δt in DMSO-*d*_6_ = −1.1 ppb/K, indicating a plausible conformation having this amide proton involved in a hydrogen bond.

**Figure 5:**
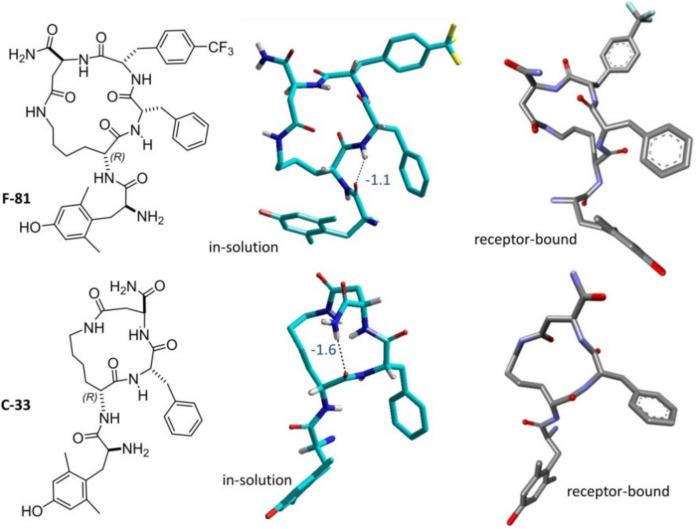
Comparison between the sketches of F-81 and C-33 and the respective in-solution structures (C is rendered in cyan, N in blue, and O in red; hydrogen bonds and the VT NMR Δδ/Δt values in ppb/K of relevant amide protons are also given) as determined by NMR analysis and molecular dynamics, compatible with VT NMR data, and the calculated receptor-bound structures (C is rendered in grey, N in blue, and O in red).

The two compounds were analyzed by 2D-ROESY in 8:2 [D6]DMSO/H_2_O, and the resulting cross-peaks were ranked by their intensity to deduce the interproton distances. Compared to F-81, C-33 showed a higher number of inter-residue proton-proton correlations, despite of the smaller size of the former. The estimated distances were analyzed by simulated annealing and restrained molecular dynamics simulations using the AMBER force field [52] in explicit water as a solvent. In brief, random geometries of each peptide were obtained by high-temperature unrestrained molecular dynamics simulation in a box of standard TIP3P models of equilibrated water [53]. For each random structure, the interproton distances were constrained at the distances inferred by ROESY. Only ROESY-derived constraints were included in the restrained molecular dynamics. Then, the structures were subjected to high temperature restrained molecular dynamics with a scaled force field, followed by a simulation period with full restraints. After slowly cooling the boxes, the geometries were minimized, and the backbones of the peptides were clustered by the root-mean-square deviation (rmsd) analysis. In all cases, this procedure gave one major cluster comprising the majority of the structures. The representative conformers with the lowest energy are reported in Figure 5.

The structure of F-81 appeared characterized by a inverse γ-turn centered on the *p*CF_3_Phe residue, and a regular γ -turn centered on d-Lys, stabilized by an explicit hydrogen bond between DmtC=O and PheNH, consistent with the Δδ/Δt parameters calculated by VT NMR (Table 3). As for C-33, the ROESY-derived structure showed a 10-membered pseudo type I β-turn closed by an explicit hydrogen bond between d-LysC=O and AspCONH_2_, in agreement to the VT NMR temperature coefficient (Table 3).

To simulate the dynamic behavior of the peptides, the ROESY-derived conformers were analyzed by unrestrained molecular dynamics in explicit water, for 10 ns at 300 K. During the simulations, the geometry of the cyclic backbone C-33 appeared more stable as compared to F-81, plausibly correlated to the smaller size of the macrolactam ring. As expected, for F-81, the simulations showed a noteworthy conformational freedom in the Dmt-d-Lys portion. The analysis of the trajectories showed the occasional formation of alternative secondary structures, stabilized by intramolecular hydrogen-bonds, not supported by the VT NMR data.

### 2.3. Molecular Docking

Docking simulations were performed with Autodock 4.0 using the receptor model derived from the 3.5 Å resolution cryo-electron microscopy structure of MOR bound to the agonist peptide DAMGO, and nucleotide-free Gi (PDB ID: 6DDF) [54]. The receptor-bound ligand structures were obtained by a systematic conformer search, followed by geometry optimization in explicit TIP3P water molecules [55]. For each ligand, 250 independent docking runs were carried out and the results were scored and clustered. The poses thus obtained were equilibrated by 1 μs molecular dynamics then, the structures of the ligands and of all the residues of the receptor were minimized by molecular mechanics.

The best-scoring poses of the two peptides are shown in Figure 5, for comparison with the in-solution conformations determined by NMR and molecular dynamics. Roughly, C-33 maintains at the receptor a conformation similar to that in solution, plausibly due its smaller, more rigid macrocycle scaffold. In contrast the receptor-bound geometry of F-81 appears quite different from the starting structure, consistent with the higher flexibility of the Dmt-Lys portion, as discussed by conformational analysis.

**Figure 6:**
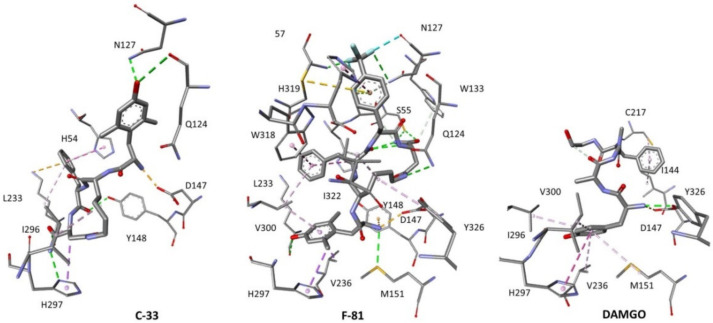
Side views of the predicted binding modes for peptides C-33, F-81, to the deposited structure of MOP (PDB ID: 6DDF), for comparison, the pose of DAMGO extracted from 6DDF is also shown [54]. The relevant receptor residue side chains are rendered in thick lines and the ligands in sticks; C is rendered in grey, N in blue, and O in red. Dashed green lines represent conventional hydrogen bonds, while cation–π interactions are rendered in yellow, π–π interactions in violet, hydrophobic interactions in white, and other interactions in pink.

The molecular modelling protocol predicted important differences in how F-81 or C-33 plausibly interact with the MOP binding pocket (Figure 6; for an alternative rear view, see the Supporting Information, Figure S9). In the complex F-81-MOR (Ballesteros-Weinstein numbering in brackets), the protonated amine of Dmt strongly interacts with residues of TM3, i.e., Asp147(3.32)CO_2_^−^ by ionic bond, Tyr148(3.33) by cation-π interaction, and is H-bonded to Met151(3.36). The phenol side chain appears well inserted within TM5 and TM6, with a hydrogen bond between phenolOH and Lys233(5.39)C=O(TM5), and hydrophobic contacts with Val236(5.42)(TM5) and His297(6.52)(TM6). The macrocycle scaffold of F-81 occupies the space between the TM helices 2, 3, and 7. The Lys linker is involved in hydrophobic contacts with Tyr326(7.43) and Ile322(7.39) of TM7, and LysNHε is H-bonded with Gln124(2.60)(TM2). The CONH_2_ of F-81 shows a H-bond with Cys217SH(EL2), and a polar contact with Ser55(N-end). The PheC=O establishes a H-bond with imidazoleH5 of His45(N-end), while the phenyl group of Phe appears sandwiched between Trp318(7.35)(TM7) and His54(N-end), due to π–π interactions, plus hydrophobic interaction with Val300(6.55)(TM6). The aryl ring of *p*CF_3_-Ph participates in several interactions. A π-lone pair interaction with Ser55C=O(N-end), a π–π interaction with His319(7.36)(TM7), a π-S interaction with Cys-S-acetamide57(YCM, N-end), and a π-donor-H-bond with Asn127(2.63)CONH_2_(TM2). Finally, the *p*CF_3_-Ph side chain is involved in a conventional F-H-bond with YCM57C=O(N-end), another conventional F-H bond with imidazoleNHτof His319(7.36)(TM7), a F-H bond with imidazoleH2 of His319(7.36), F-H bond with CHβ of Tyr128(2.64)(TM2), and in halogen-bond interactions with Ser55C=O(N-end), Leu56C=O(N-end), Asn127(2.63)C=O (TM2).

The calculated structure of C-33-MOP appeared clearly different from the one discussed above. The CONH_2_ group of Asp is found deeply buried into the inner cavity delimited by TM5 and TM6. Besides, the entire Dmt residue is rotated in the opposite direction (Figure 6), pointing towards the extracellular rim of the receptor. Albeit infrequent, a similarly reversed position of the tyramine residue was observed in cyclic opioid peptides [56,57].

Nevertheless, the quaternary ammonium cation of Dmt is still capable of interaction with the Asp147(3.32)CO_2_^−^ (TM3) via ionic bond. Dmt is also kept in place by H-bonds involving phenol oxygen and TM2, with Asn127(2.63)CONH_2_ and Gln124(2.60)Hα. The macrocycle of C-33 is lodged within the cavity delimited by helices 3 and 5–7. LysNH shows a H-bond with His54NHτ(N-end), while its C chain displays a hydrophobic interaction with Ile296(6.51) (TM6). AspHβ shows a π-Hα interaction with His297(6.52)(TM6). The amide H of CONH_2_ is linked to His297(6.52)Nπ (TM6) with a strong H-bond (1.89 Å), while the oxygen of CONH_2_ is involved in an interaction with Lys233(5.39)Hα (TM5). As for Phe residue, a H-bond can be detected between its C=O and Tyr148OH(3.33)(TM3), while the aryl ring makes a π-cation interaction with Lys233(5.39)NH_3_^+^(TM5), plus hydrophobic contacts with the C chain of the same residue, and both π–π and π-NH interactions with His54(N-end).

**Figure 7:**
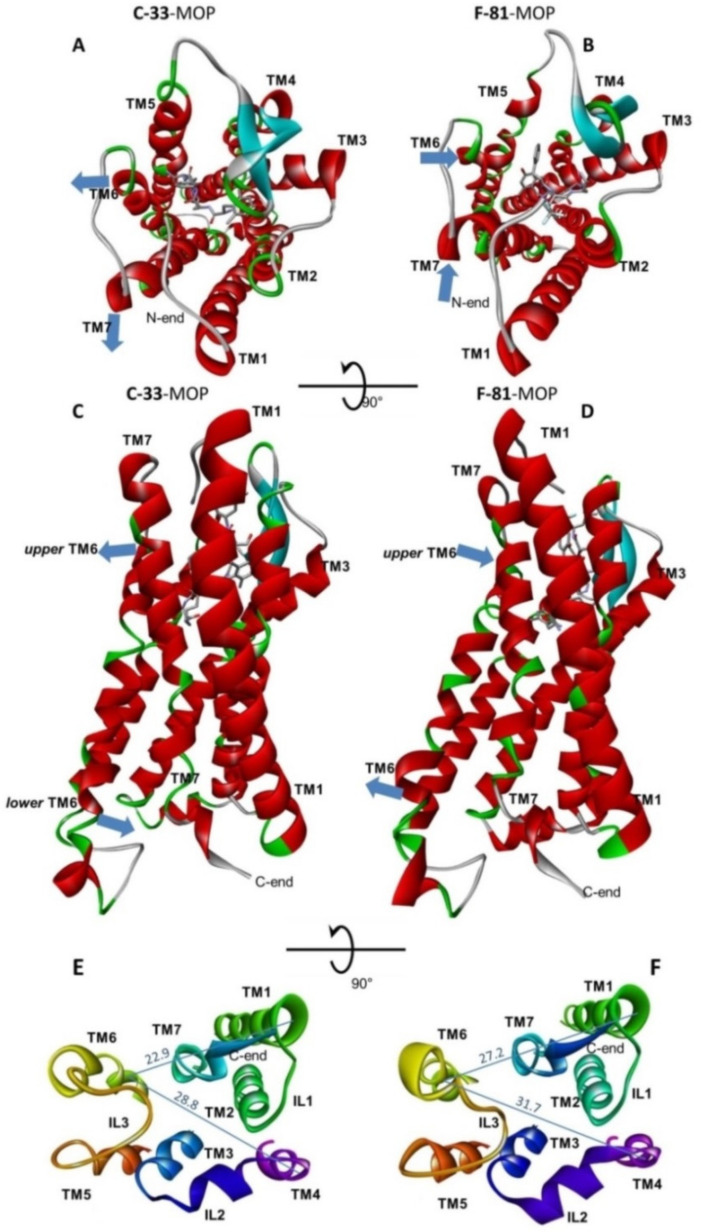
Views of the predicted binding poses of C-33-MOP and F-81-MOP. (**A**) Top views of C-33-MOP and (**B**) of F-81-MOP, showing in particular the different position of TM6 and TM7. (**C**) Side views of C-33-MOP and (**D**) of F-81-MOP, highlighting the inclination of TM6. (**E**) cytosolic ends of the helices and the intracellular loops of C-33-MOP and (**F**) of F-81-MOP; the distances between the Cαs of Arg280(TM6)-Arg182(TM4) and Arg280-Tyr91(TM1) are given in Å (these residues have been selected arbitrarily for easy comparison). The cyan arrows indicate the comparatively diverse positions of TM6 and TM7 of the two complexes.

To summarize, the modeling computations suggested that two ligands establish different networks of interactions with the receptor cavity. Interestingly, the inspection of the complexes F-81-MOP and C-33-MOP predicted noteworthy differences also in the relative positions of the helices. In particular, the TM6 of C-33-MOP appears shifted inwards compared to F-81 (Figure 7A), while the remaining helices maintain circa the same positions.

## 3. Discussion

Opioids are the most effective treatments for acute and chronic pain, yet their use is hampered by unpleasant and dangerous side effects, including lethal overdose. Understanding opioid receptor activation mechanism and their signaling pathways might allow creation of safer and more effective treatments. Experiments conducted with β-arrestin 2 knockout animals indicated that MOP activation produced analgesia, but significantly fewer side effects [9]. Therefore, significant efforts have been placed on designing ligands biased toward G-protein activation [17,18,19,20,21,22,23,24,25,26,27,28], a requirement for analgesic efficacy, while avoiding interaction with β-arrestin. Biased ligands are believed to stabilize specific receptor conformations distinct from those of unbiased agonists [15,16].

Nevertheless, the mechanism of action and the potential clinical utility of functionally selective agonists at opioid receptors is still lively debated. Recent in vivo data obtained using phosphorylation-deficient MOP [33] showed that total abolishment of β-arrestin binding improved analgesia and diminished tolerance, but worsened opioid side effects. Besides, studies on D2 dopamine GPCR showed that agonist-dependent signaling bias was linked to the binding kinetics of the agonists [41,42]. This led some authors to ask the question whether GPCR bias is driven by kinetics and cellular background rather than conformation.

However, recent work revealed that for MOP biased agonism of pharmacologically heterogeneous agonists is not controlled by binding and signaling kinetics, corroborating a mechanism driven by receptor conformations [43]. Structural and biophysical studies have begun to clarify from a mechanistic perspective how GPCRs mediate biased signaling [58]. In the antagonist-bound state, GPCRs assume an inactive conformation with the cytoplasmic ends of the helices packed with each other, thus blocking the interactions of any effectors [59]. Upon binding, an agonist promotes a massive movement of the cytoplasmic side of the receptor [60]. In particular, the active conformation of MOP seems to be characterized by an outward displacement of TM6 from the heptahelical bundle relative to the inactive state [61]. It has been proposed that arrestin-biased drugs are able to stabilize a distinct receptor conformation in which motifs that are essential for arrestin-biased signaling are activated, while others associated with G protein signaling remain in the inactive state [16]. The comparison of the crystal structures suggested that the outward displacement of TM6 is more pronounced in rhodopsin-G than rhodopsin–arrestin complex [62,63].

In this perspective, we investigated the cyclopeptide MOP ligands F-81 and C-33 (Figure 1), indicated by preliminary data as biased activators of G protein and β-arrestin 2 signaling, respectively, as determined by the calcium mobilization assay and the BRET assay. New experiments described herein substantiated the supposed, biased profile of the two compounds. In the [^35^S]GTPγS assay (Figure 2), F-81 and C-33 both stimulated MOP with similar potency, and F-81 was a high efficacy partial agonist (Table 1). In the PathHunter enzyme complementation assay (Figure 3), the two compounds stimulated the β-arrestin 2 signaling pathway, with F-81 being low efficacy partial agonist with higher than C-33 potency, and C-33 displaying higher efficacy than the reference EM-2 (Table 3).

Since the MOP agonists F-81 and C-33 showed a certain preference for G protein or β-arrestin 2 signaling, respectively, we utilized these two peptides as scaffolds for the computational investigation of the structural determinants of their biased signaling. Initially, we determined the in-solution conformations of both compounds by NMR spectroscopy (Figure 4), molecular modeling and molecular dynamics (Figure 5), then we performed a molecular docking and molecular dynamics simulations of the peptide-MOP complexes. The results are shown in Figure 6 and Figure 7, which predict distinct ligand–receptor interactions and conformations that could underlie the differences in bias.

Figure 7A,B show the top views of C-33-MOP and F-81-MOP, respectively. The more relevant differences can be perceived in the position of the extracellular ends of TM6 and TM7. In the complex F-81-MOP, the ligand occupies the center of the cavity delimited by the helices 2–7, while in C-33-MOP the ligand leans against TM6 and TM7, this plausibly producing the outward positioning of these two helices (Figure 7A, cyan arrows).

The significant push exerted by C-33 against the upper part of TM6 seems compatible with the relevant contacts between this ligand and TM6 (Figure 6 and Figure S10, Supporting Information). Indeed, C-33 was found to interact with TM6 by means of a strong H-bond between its CONH_2_ side chain and the imidazole of His297(6.52), plus hydrophobic interactions between the macrolactam scaffold and Ile296(6.51), Val300(6.55) (Figure S10), Supporting Information.

As for peptide F-81, few interactions have been detected with TM6. The Dmt residue adopts a pose very similar to that shown by the tyramine of DAMGO in the MOP-Gi protein complex (Figure 6) obtained by cryo-electron microscopy [54], albeit lacking of direct interactions between Dmt and TM6. This is in contrast to the structure of DAMGO-MOP, since the latter, after refinement by molecular dynamics simulations, predict a contact between phenolic-OH and the imidazole of His297(6.52) (TM6) mediated by one water molecule. For few other opioid peptides reported in the literature, molecular modeling suggested a direct H-bond interaction between the phenol and the imidazole of His297(6.52) [64].

The side views of C-33-MOP and F-81-MOP show that TM6 acts as a rigid body (Figure 7C,D), since the lower half of this helix follows the rotation of the upper part, anticlockwise in C-33-MOP (Figure 7C, cyan arrows) and clockwise in F-81-MOP (Figure 7D). In contrast, the lower half of TM7 circa maintains the same position in both complexes.

The Figure 7E,F show the cytosolic ends of the helixes for the two complexes, as determined by molecular dynamics and energy minimization for the whole complexes. Clearly, the contraction of TM6 in C-33-MOP results in a smaller cavity between the helices TM2, TM3, and TM5-TM7. This is consistent with the proposed binding pocket for arrestins at the base of the rhodopsin being slightly smaller than those for G proteins [26], and is consistent with the arrestin-biased profile of C-33 [62,63].

**Figure 8:**
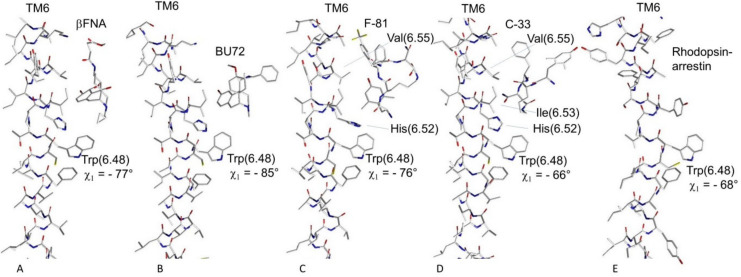
Side views of TM6 as extracted from the complexes of MOP with (**A**) the antagonist βFNA [59], (**B**) the G-protein biased agonists BU72 [61] and (**C**) F-81, (**D**) the β-arrestin biased agonist C-33, and (**E**) from the rhodopsin–arrestin complex [63].

Overall, our simulations support the role of TM6 in receptor activation, in line with the evidence from crystallographic data [59,60,61]. However, the precise mechanism underlying the transmission of conformational changes along TM6 is still poorly understood. The comparison of the crystal structures of the complexes between MOP and the antagonist β-funaltrexamine (βFNA) [59] and the agonist BU72 (Figure 1) [61] showed that the morphinan scaffolds were positioned differently in the inactive and active structures. During the conformational transition from inactive to active receptor, there is a rearrangement of the amino acids that lay just below the binding pocket. This rearrangement strongly impacts the residues Phe289(6.44) and Trp293(6.48). In particular, the latter seems the most responsible in the propagation of conformational changes. Agonists would stabilize Trp293(6.48) in the rotamer observed in the active-state crystal structure (Figure 8), thus inducing the outward positioning of this helix, and hence to the open, effector-accessible conformation of the intracellular portions of the helices.

Very recently, Mafi et al. analyzed the structures of activated β-arrestin 2 stabilized by phosphorylated MOP bound to the morphine and DAMGO nonbiased agonists and to the TRV130 biased agonist, in comparison with the complexes of the same ligands with G-protein. Extensive molecular dynamics simulations for biased and nonbiased ligands showed that Gi protein and β-arrestin 2 compete for the same binding site. However, the peculiar binding pose of the G-protein biased TRV130 appeared to reposition TM6 in the cytoplasmic region, hindering β-arrestin 2 from making polar anchors to the ICL3 or to the cytosolic end of TM6. This seemed to dramatically reduce the affinity between MOP and β-arrestin 2 [65].

Molecular dynamics simulations confirmed the role of Trp293(6.48) as the most responsible in the propagation of conformational changes of TM6 and in general of the whole receptor. Simulations of the β-arrestin bias agonist fentanyl-MOP complexes were studied using both inactive and active opioid receptor crystal structures, in comparison to the unbiased morphine. Fentanyl stabilizes different rotameric states of Trp293(6.48) than observed for morphine. Morphine was able to contact five TM6 residues, and fentanyl only three. The dihedral angles in Phe289(6.44) and Trp293(6.48) appeared to be correlated. In particular, Trp293(6.48) was thought to be directly involved in the activation process, whose rotation would open the space toward the cell interior [66].

Together with the residues of TM6, other residues of MOP have been proposed to cooperate in biased receptor activation. Computations on TRV-130 in the activated MOP crystal structure investigated the complex, multistep process of ligand approach to the receptor after establishing the first contact, the residence in the vestibule, and the eventual penetration in the orthosteric site. The results suggest that Trp293(6.48) and Tyr326(7.43) function as both stabilizers of the dynamics of the binding pocket, and communicators that maximally contribute to the coupling between the binding pocket and the intracellular region of the receptor [67].

De Waal et al. studied the mechanism of MOP recognition and activation for fentanyl. Following in-silico computations accelerated by the use of mollified adaptive biasing potential, they proposed a mechanism where fentanyl mediates MOP β-arrestin through a peculiar microswitch of the position of Met(3.36). This residue is pushed downward by fentanyl’s n-aniline ring, to adopt a rotameric conformation which directly displaces Trp(6.48) [68].

Cheng et al. constructed five MOP systems in complexes with the G-protein-biased agonists TRV130 and BU72, the antagonists β-FNA and naltrexone, as well as the free receptor. Molecular dynamics and analyses of G-protein-biased activation and inactivation mechanisms pointed to an activation switch composed of residues Trp293(6.48) and Trp326(7.43). In the TRV130 system, Trp293(6.48) was more stable than in the other systems, and the torsion angle of its side chain was mostly stabilized at −70° [69].

Subsequent molecular dynamics simulation of MOP upon the binding of morphine, TRV130, and PZM21, were performed [70], aimed at determining the causes of low-potency and lower bias effects of PZM21 compared with the other two ligands [32]. Differences in conformational changes of Trp318(7.35), Tyr326(7.43), and Tyr336(7.53) in PZM21 and TRV130 explained the observed differences in bias between these ligands. Eventually, the authors deduced that the delayed movement of the Trp293(6.48) side chain was a factor determining the dose-dependent agonism of PZM21.

Owing to the many hints about the role of Trp293(6.48) as “activation switch”, as discussed above, we analyzed in detail its rotameric states in the complexes C-33-MOP and F-81-MOP, in comparison with the geometries extracted from the crystal structures of the complexes of MOP with the antagonist βFNA [59], the G-protein biased agonists BU72 [61], and in the rhodopsin–arrestin complex [63] (Figure 8).

The inspection of the structures depicted in Figure 8 shows slightly diverse displays of the residues of TM6 beneath the ligand-binding site. As recalled above, antagonists or agonists stabilize Trp293(6.48) in diverse rotamer in the inactive- (Figure 8A) or active-state crystal structure (Figure 8B). For the cyclopeptides C-33-MOP and F-81-MOP, it can be supposed that the diverse interaction patterns of the ligands with the neighboring residues of TM6 might in turn affect the conformational state of Trp293(6.48). In the F-81-MOP complex, the side chain of Trp293(6.48) shows a χ_1_ value of −76° (Figure 8C). Remarkably, the arrestin-biased peptide C-33 seems to stabilize a torsion angle χ_1_ of the indolyl side chain of −66° (Figure 8D), which appears nicely consistent to the χ_1_ value of −68° as observed in the rhodopsin–arrestin complex (Figure 8E).

## 4. Materials and Methods

*Peptide synthesis.* C-33 and F-81 were prepared as C-terminal amides by standard solid-phase synthesis on MBHA resin (100–200 mesh, 0.8 mM/g), using the 9-fluorenylmethoxycarbonyl (Fmoc) strategy, and 2-(1*H*-benzotriazol-1-yl)-1,1,3,3-tetramethyluronium tetrafluoroborate (TBTU) and diisopropylethylamine (DIEA) as the coupling agents. d-Lys was introduced as Fmoc-d-Lys(Mtt)-OH (Mtt, ε-4-methyltrityl), Asp was introduced as Fmoc-Asp(*O*-2-PhiPr)-OH (*O*-2-Ph*i*Pr, β-phenyl-isopropyl ester), and Dmt as Fmoc-Dmt(*t*-Bu)-OH. After each coupling, the Fmoc protecting group was removed with 20% (*v*/*v*) piperidine in dimethylformamide (DMF), in 20 min. After synthesis, the side-chain groups Mtt and *O*-2-Ph*i*Pr were removed by on-resin treatment with 1% *v*/*v* trifluoroacetic acid (TFA) in dichloromethane (DCM), followed by on-resin cyclization with TBTU and DIEA. Then, the N-terminal Fmoc group was removed with piperidine/DMF, and the cyclized peptides were cleaved from the resin with a mixture of TFA/triisopropylsilane/water (95:2.5:2.5, *v*/*v*), during 3 h at rt. The crude peptides were precipitates in ice-colt ether, and were purified by RP-HPLC on a Vydac C_18_ column (22 mm by 250 mm, 10 µm) using a linear gradient of mobile phase from 100% A to 100% B over 15 min, at the flow rate of 2 mL/min; A, 0.1% TFA in water and B, 0.1% TFA in acetonitrile/water (80:20, *v*/*v*). The purity of the final peptides was assessed by analytical RP-HPLC on a Vydac C_18_ column (4.6 by 250 mm, 5 µm,) using the same solvent system over 50 min at a flow rate of 1 mL/min. The peptides were characterized by NMR and ESI-MS (Supplementary Information).

*[^35^S]GTPγS binding assay.* CHO cells stably expressing the human MOP receptor (in brief, CHO-hMOP cells, provided by Prof G. Calo, University of Ferrara; originator was Prof L. Toll, SRI International, Menlo Park, CA, USA) were grown in Hams F12 containing streptomycin (100 μg/mL), fungizone 2.5 μg/mL), penicillin (100 IU/mL) and 10% fetal bovine serum (FBS). Stock medium containing G418 (200 μg/mL) was used to maintain CHO-hMOP expression. Experiments were performed according to the literature [46,47]. Cell membranes (5–10 µg) were incubated at 30 °C with 0.05 nM [^35^S]GTPγS (PekinElmer, 549 Albany St, Boston, MA 02118, US), 10 µM GDP and various concentrations of a tested peptide in the final volume of 1 mL, for 60 min. Non-specific binding was determined using 10 µM GTPγS, and the basal binding was determined in the absence of a tested peptide. Incubations were terminated by rapid filtration through the GF/B Whatman glass fiber strips and the bound radioactivity was measured in a liquid scintillation counter. Five independent experiments were carried out. The data were analyzed by a nonlinear least square regression analysis computer program Graph Pad PRISM 5.0 (GraphPad Software Inc., San Diego, CA, USA).

*Measurement of β-arrestin 2 recruitment.* β-arrestin 2 recruitment at MOP was measured using the DiscoveRxPathHunter^®^ β-arrestin kit (DiscoveRx, Fremont, CA, USA) according to manufacturer’s recommendations [49] and previously published procedure [38]. CHO cells were engineered to co-express the ProLink™ (PK) tagged GPCR (GPCR-PK) and the enzyme acceptor (EA) tagged β-arrestin 2 (β-arrestin-EA) (CHO-K1 OPRM1 β-arrestin cell line). Activation of the GPCR-PK induces β-arrestin-EA recruitment, forcing complementation of the two β-galactosidase enzyme fragments (EA and PK). The resulting functional enzyme hydrolyzes substrate to generate a chemiluminescent signal which was measured by luminometry. Briefly, 20 μL of PathHunter cells as taken from the kit were seeded in each well and incubated overnight at 37 °C. Agonist solution in DMSO (5 µL; the maximum concentration of DMSO was 0.005%, then serially diluted 1:10; we have previously determined that this ultra-low concentration does not affect binding so no additional control was included) was added and the cells were incubated for 90 min at 37 °C. Detection Reagent Working Solution (12 μL) was added and incubation was continued for 60 min at rt. Chemiluminescent signal was read using a Viewlux imaging plate reader (PerkinElmer, Cambridge, MA, USA).

*NMR analysis. *^1^H-NMR spectra were recorded on a 400 MHz Varian instrument in 5 mm tubes, in DMSO-*d*_6_ or 8:2 DMSO-*d*_6_/H_2_O, at 0.01 M peptide concentration, using residual DMSO δH = 2.50 ppm as internal standard; ^13^C-NMR were recorded at 100 MHz, using residual DMSO δC = 39.5 ppm as internal standard. The unambiguous assignment of ^1^H-NMR resonances was based on 2D gCOSY experiments. Water suppression was performed by the solvent presaturation procedure implemented in Varian (PRESAT). Variable temperature (VT)-^1^H-NMR experiments were performed over the range of 298–348 K; temperature calibration was done with the ethylene glycol OHCHn chemical-shift separation method.

F-81. ^1^H-NMR (8:2 DMSO-*d*_6_/H_2_O, 400 MHz): δ = 0.71 (m, 1H, LysHγ), 0.94 (m, 1H, LysHγ), 1.12–1.29 (m, 4H, LysHβ + δ), 2.18 (s, 6H, DmtMe), 2.35–2.55 (m, 2H, AspHβ), 2.74 (dd, *J* = 5.6, 13.6 Hz, 1H, PheHβ), 2.82–3.05 (m, 5H, 2×DmtHβ + 1 × CF_3_PheHβ + 1 × LysHε + 1 × PheHβ), 3.06 (m, 1H, LysHε), 3.17 (dd, *J* = 4.8, 13.6 Hz, 1H, CF_3_PheHβ), 3.79 (m, 1H, DmtHα), 4.02 (m, 1H, PheHα), 4.15 (m, 1H, LysHα), 4.45 (m, 1H, CF_3_PheHα), 4.48 (m, 1H, AspHα), 6.42 (s, 2H, DmtArH), 7.03 (d, *J* = 6.8 Hz, 2H, PheArH), 7.07–7.17 (m, 2H, CONH_2_), 7.17–7.26 (m, 3H, PheArH), 7.37 (d, *J* = 7.6 Hz, 2H, CF_3_PheArH), 7.60 (d, *J* = 7.6 Hz, 2H, CF_3_PheArH), 7.66 (br.t, 1H, CONHε), 7.82 (br.d, 1H, LysNH), 7.85 (br.d, 1H, CF_3_PheNH), 7.89 (br.d, 1H, PheNH), 8.09 (d, *J* = 8.4 Hz, 1H, AspNH), 8.20–8.50 (m, 3H, DmtNH), 9.09 (s, 1H, DmtOH); ^13^C-NMR (DMSO-*d*_6_, 100 MHz): δ = 20.4, 21.8, 28.5, 31.2, 31.5, 36.8, 37.0, 38.5, 38.8, 50.4, 52.0, 53.1, 54.6, 57.6, 115.4, 122.3, 125.3, 126.8, 128.6, 129.4, 130.5, 137.8, 138.7, 143.2, 156.3, 168.5, 169.6, 170.3, 171.2, 172.1, 172.1, 172.9.

C-33. ^1^H-NMR (8:2 DMSO-*d*_6_/H_2_O, 400 MHz): δ = 0.70 (m, 1H, LysHγ), 0.87 (m, 1H, LysHγ), 1.04 (m, 1H, LysHβ), 1.14 (m, 1H, LysHδ), 1.25–1.38 (m, 2H, LysHδ+LysHβ), 2.17 (s, 6H, DmtMe), 2.66 (dd, *J* = 12.0, 14.0 Hz, 1H, AspHβ), 2.42 (dd, *J* = 1.6, 14.0 Hz, 1H, AspHβ), 2.61 (dd, *J* = 6.4, 13.6 Hz, 1H, PheHβ), 2.75 (m, 1H, LysHε), 2.90 (dd, *J* = 4.4, 14.0 Hz, 1H, DmtHβ), 2.97 (dd, *J* = 11.2, 14.0 Hz, 1H, DmtHβ), 3.04 (dd, *J* = 8.4, 13.6 Hz, 1H, PheHβ), 3.35 (m, 1H, LysHε), 3.78 (m, 1H, TyrHα), 4.12 (m, 1H, LysHα), 4.53 (m, 1H, AspHα), 4.59 (m, 1H, PheHα), 6.44 (s, 2H, DmtArH), 6.56 (s, 1H, CONH_2_), 7.00 (s, 1H, CONH_2_), 7.10–7.19 (m, 3H, PheArH), 7.19–7.26 (m, 2H, PheArH), 7.45 (m, 1H, CONHε), 7.65 (d, *J* = 8.0 Hz, 1H, LysNH), 7.73 (d, *J* = 8.8 Hz, 1H, PheNH), 7.80 (d, *J* = 8.4 Hz, 1H, AspNH), 8.27–8.40 (m, 3H, DmtNH), 9.15 (s, 1H, DmtOH); ^13^C-NMR (DMSO-*d*_6_, 100 MHz): δ = 20.3, 28.9, 30.8, 31.7, 36.8, 37.4, 38.3, 49.0, 50.8, 52.1, 53.7, 53.8, 115.3, 122.4, 126.8, 128.7, 129.3, 138.0, 138.7, 156.2, 168.3, 169.5, 170.4, 170.5, 173.6.

*Conformational analysis* [71]. 2D ROESY experiments were performed at rt in the phase-sensitive mode, with a spin-locking field (γb2) of 2000 Hz, and a mixing time of 250 ms. Spectra were processed in the hypercomplex approach, and peaks were calibrated on solvent. H-bond interactions and torsion angle restraints were not utilized. For the absence of Hα*i*-Hα*(i + 1)* cross-peaks, reasonably excluding the occurrence of cis peptide bonds, all ω bonds were restricted at 180° with a force constant of 16 kcal mol^−1^ Å^−2^. Cross-peak intensities were associated to the following distances, in Å: very strong = 2.3, strong = 2.6, medium = 3.0, and weak = 5.0. The intensities of geminal protons were found to match with these associations, but were not utilized.

*Simulated annealing*. Restrained MD simulations [72] were conducted at 1 atm with the AMBER force field [52] in a 30Å by 30Å by 30Å box of standard TIP3P models of equilibrated water [53], periodic boundary conditions, a dielectric scale factor of 1, and cutoff for the nonbonded interactions of 12 Å. The water molecules closer than 2.3 Å to the peptide atoms were deleted. During a 100 ps simulation at 1200 K, 50 structures were randomly selected. Each structure was subjected to a restrained MD for 50 ps at 1200 K, applying a 50% scaled force field, then by a 50 ps MD with full distance restraints, applying a force constant of 7 kcal mol^−1^ Å^−2^, then the system was cooled to 50 K during 20 ps. The resulting structures were minimized by 3000 cycles of steepest descent and 3000 cycles of conjugated gradient, with a convergence limit of 0.01 kcal Å^−1^ mol^−1^. The backbones of the structures were clustered by the rmsd analysis.

*Unrestrained molecular dynamics.* The simulations were performed in the 30 Å by 30 Å by 30 Å box of standard TIP3P water for 10 ns at 298 K using periodic boundary conditions, at constant temperature and pressure with the Berendsen scheme [73], and a bath relaxation constant of 0.2. The 1–4 scale factors, van der Waals, and electrostatic interactions, are scaled to half their nominal value in AMBER. The integration time step was set to 0.1 fs. The coordinates of the system were collected every 1 × 10^−12^ s.

*Molecular docking.* The MOP model in its active conformation was extracted from the deposited X-ray structures (PDB ID: 6DDF). The structures of the ligands were subjected to systematic conformer search followed by geometry optimization to the lowest energy structure with MOPAC7 (PM3, RMS gradient 0.01). Hydrogen atoms were added with respect of H-bonding network by Reduce [74]; PROPKA [75] was employed to estimate the protonation states of the titratable residues. Molecular docking was performed with Autodock 4.0 [76] with the Lamarckian Genetic Algorithm; global search utilized the Genetic Algorithm alone; local search utilized the Solis and Wets algorithm [77]. Ligands and receptors were handled using the Autodock Tool Kit (ADT) [78]. Gasteiger–Marsili charges [79] were added to the ligands in ADT and solvation parameters were introduced to the final structure by Autodock Addsol utility. Each docking run involved an initial population of 250 random geometries, with a limit of 500 energy evaluations, mutation rate = 0.02, crossover rate = 0.80, elitism = 1.250 iterations were performed per each local search (250 independent docking runs for each ligand). The grid maps representing the system in the actual docking process were calculated with Autogrid, and the docking results were scored by the inter-molecular energy function based on the simple Weiner force field in Autodock. The structures were clustered when the rmsd was less than 1.0 Å, and the clusters were represented by the structure with the lowest free energy of binding. The poses thus obtained were equilibrated by a 5 ns partially restrained molecular dynamics simulation. The complexes were then embedded in a lipid and cholesterol bilayer [26], and used in all-atom unrestrained molecular dynamics for 1 μs at 300 K, using the CUDA^®^ version of the GROMACS package [80] with a modified version of the AMBER ff03 force field [81], a variant of the AMBER ff991 potential in which charges and main-chain torsion potentials have been derived based on QM + continuum solvent calculations and each amino acid is allowed unique main-chain charges. Unless otherwise noted, simulations were carried out in the isochoric isothermal (NVT) ensemble at 303.15 K using a Langevin thermostat [82] with a 2 fs timestep and a friction constant of 0.5 ps^−1^ applied to all atoms. All bonds to hydrogen atoms were constrained using the LINCS algorithm [83]. After the above-described MD simulations, the complexes were subjected to an Amber ff99 force field [84]. Non-bonded interactions were calculated with a cutoff of 10 Å. The calculated Autodock binding scores in kcal mol^−1^ were −12.99 for C-33, and −12.09 for F-81.

## 5. Conclusions

Currently, much effort is being dedicated to clarifying the pharmacological effects of biased agonists of the opioid receptors and other GPCRs. Hence, the identification and investigation of biased ligands has become the object of increasing interest. Biased ligands are believed to stabilize distinct receptor conformations, allowing for the recruitment of diverse intracellular effectors. Recently, we obtained the cyclopeptides F-81 and C-33, characterized by high affinity for MOP, which showed to preferentially activate G or β-arrestin signaling, respectively, as determined by calcium mobilization assay, BRET assay, and by [^35^S]GTPγS binding assay, and the PathHunter enzyme complementation assay. It is our opinion that the MOP agonists F-81 and C-33 might contribute to the debate about the origins of biased agonism. Conformational analysis, molecular docking, and dynamics simulations of the complexes of F-81 and C-33 with MOP, predicted distinct ligand–receptor interactions and conformations that could explain the differences in bias [58,59,60,61,62,63]. The more flexible peptide F-81 seems to adopt a “classic” pose in the peptide-MOP complex, and establishes intermolecular interactions, consistent with observed G protein preference at related GPCRs. In contrast, the β-arrestin biased peptide C-33 seems forced to fit the receptor cavity by adopting a reversed pose, plausibly due to its higher conformational rigidity. The more intense contacts established with TM6 plausibly dictate the display of this helix, resulting in a more compact grouping of the cytosolic ends of the helices, as compared to F-81.

## Figures and Tables

**Table 1:** G-protein activation ([^35^S]GTPγS binding) in response to different ligands.

Compd	CHO-hMOP		
	pEC_50_	%Stim	Class
EM-1	7.45 ± 0.09	100	Full agonist
F-81	9.95 ± 0.21 *	68 ± 1 *	Partial agonist
C-33	10.10 ± 0.30 *	88 ± 3	Full agonist

Notes: Data are mean + SEM (*n* = 5). * *p* < 0.05 ANOVA with post-hoc Bonferroni, significantly different from EM-1.

**Table 2:** Ligand induced β-arrestin recruitment in CHO-K1 OPRM1 (μ-opioid receptor (MOP))/β-arrestin 2 PathHunter assay.

Compd	CHO-K1 OPRM1 β-Arrestin 2 Cell Line	
	pEC_50_	%Stim
EM-1	7.40 ± 0.16	100
F-81	9.40 ± 0.20 *	41 ± 15 **
C-33	8.60 ± 0.20 *	156 ± 3 ***

Notes: Data are mean ± SEM (*n* = 5); * *p* < 0.05 ANOVA and Bonferroni post-hoc test; more potent than EM-1. ** *p* < 0.05 ANOVA and Bonferroni post-hoc test; lower efficacy than EM-1. *** *p* < 0.05 ANOVA and Bonferroni post-hoc test; higher efficacy than EM-1.

**Table 3:** Δδ/Δt (ppb/K) values for peptides F-81 and C-33 in DMSO-*d*_6_.

Compd	d-LysNH	PheNH	CF_3_PheNH	AspNH	CONH_2_	CONHε
C-33	−3.6	−2.7	--	−4.5	−1.6/−5.0	−3.6
F-81	−3.6	−1.1	−2.1	−2.2	−4.4/−4.9	−3.5

## Supplementary Materials

The following are available online. HPLC chromatograms; high resolution MS spectra; summary of previously reported receptor affinity and functional assays; NMR spectra; ROESY cross peaks; rear views of C-33/MOP, F-81/MOP; details of the docking poses of C-33 or F-81 and TM6 [44,45].

## Sample Availability

Samples of the compounds F-81 and C-33 are available from the authors.
