# Supplementary material for: Pharmacological Characterization of µ-Opioid Receptor Agonists with Biased G Protein or β-Arrestin Signaling, and Computational Study of Conformational Changes during Receptor Activation
Source: Molecules. 2020 Dec 22;26(1):13. doi: 10.3390/molecules26010013 (PMC7792944; doi:10.3390/molecules26010013)
Supplement: Supplementary file 1 [file molecules-26-00013-s001.pdf]

Supporting information

Pharmacological characterization of μ-opioid receptor agonists showing biased G protein or β-arrestin signaling and computational study of conformational changes during receptor activation

Justyna Piekielna-Ciesielska[1], Roberto Artali[2], Ammar A.H. Azzam[3,6], David G. Lambert[3], Alicja Kluczyk[4], Luca Gentilucci[5,]*, Anna Janecka[1,]*.

[a] *Department of Biomolecular Chemistry, Medical University of Lodz, Mazowiecka 6/8, 92-215 Lodz, Poland*

[b] *Scientia Advice, di Roberto Artali, 20832, Desio, Monza and Brianza, Italy*

[c] *Department of Cardiovascular Sciences, University of Leicester, Anaesthesia, Critical Care and Pain Management, Leicester Royal Infirmary, Leicester, UK.*

[d] *Faculty of Chemistry, University of Wroclaw, F. Joliot-Curie 14, 50-383 Wroclaw, Poland*

[e] *Department of Chemistry, University of Bologna, Via Selmi 2, 40126, Bologna, Italy.*

[f] *Home address College of Pharmacy, University of Babylon, Babylon 51002, Iraq.*

* Corresponding authors: Luca Gentilucci, PhD, E-mail: luca.gentilucci@unibo.it; Anna Janecka, PhD, E-mail: anna.janecka@umed.lodz.pl

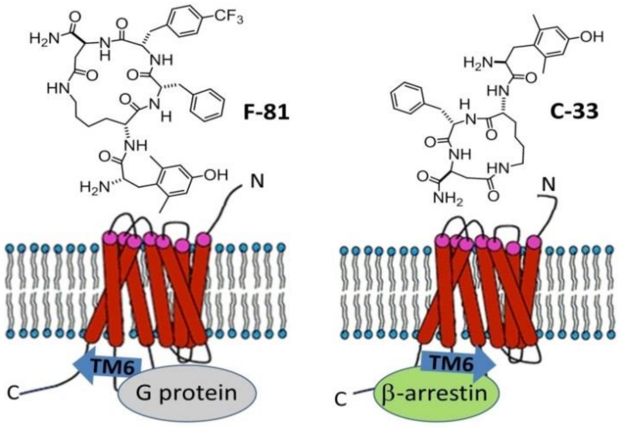

Contents

Fig. S1, S2 Analytical HPLC chromatograms …………………………….…. pS2
Table S1, Fig. S3, S4 High resolution MS spectra ……..……………………....... pS2
Fig. S5-S8 NMR spectra ……………………………………………………. pS4
Tables S2-4 ....……………………………… pS6
Tables S5, S6 Roesy cross-peaks ……………………………………….... pS7
Fig. S9 Rear views of **C-33**/MOP, **F-81**/MOP …………………………………. pS9
Fig. S10 Interactions between **C-33** or **F-81** and TM6 ……………………………….. pS9

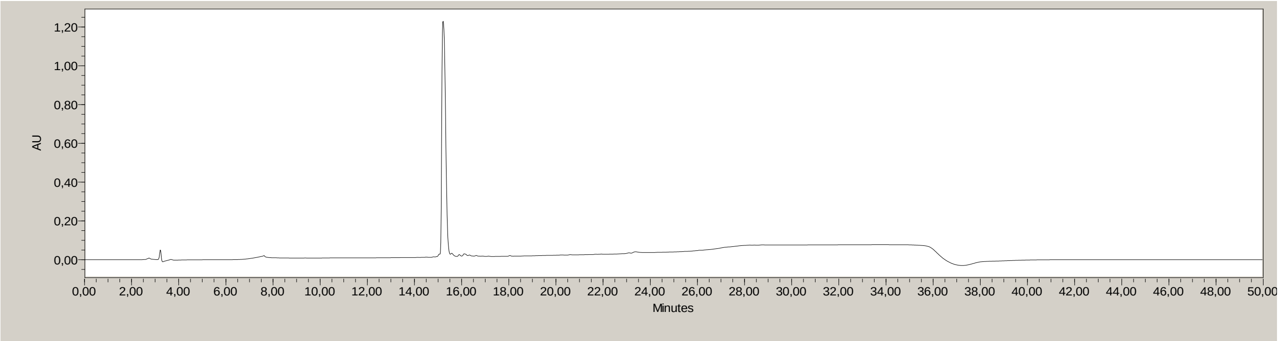

Fig. S1. Analytical HPLC chromatogram of peptide Dmt-c[D-Lys-Phe-Asp]NH_2 (**C-33**).

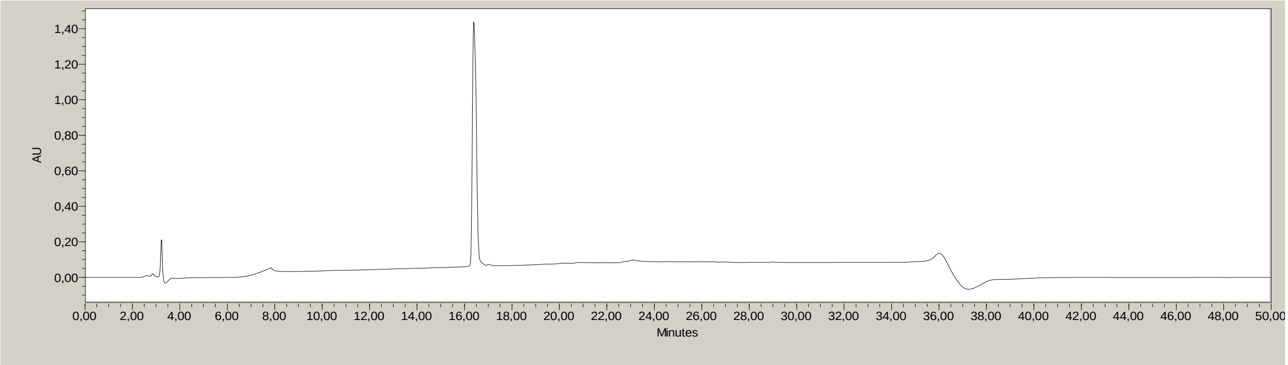

Fig. S2. Analytical HPLC chromatogram of peptide Dmt-c[D-Lys-Phe-pCF_3-Phe-Asp]NH_2 (**F-81**).

Table S1. Mass spectra of peptides were recorded using Bruker micrOTOF-Q mass spectrometer with electrospray ionization (ESI-MS) (F-**81**) or Bruker FTICR (Fourier transform ion cyclotron resonance) Apex-Qe Ultra 7 T mass spectrometer equipped with standard ESI source (C-**33**) (Bruker, Germany). The instruments were operated in the positive-ion mode and calibrated with the Tunemix™ mixture (Agilent Technologies, Palo Alto, CA, USA).

	Sequence	Molecular weight [M]	Formula for $[M+H]^+$	m/z calculated for $[M+H]^+$	m/z found $[M+H]^+$
**C-33**	Dmt-c[D-Lys-Phe-Asp]NH_2	580.6752	$C_{30}H_{41}N_6O_6$	581.3082	581.3064 FT ICR
**F-81**	Dmt-c[D-Lys-Phe-pCF_3-Phe-Asp]NH_2	795.8470	$C_{40}H_{49}F_3N_7O_7$	796.3640	796.3725 micrOTOFQ

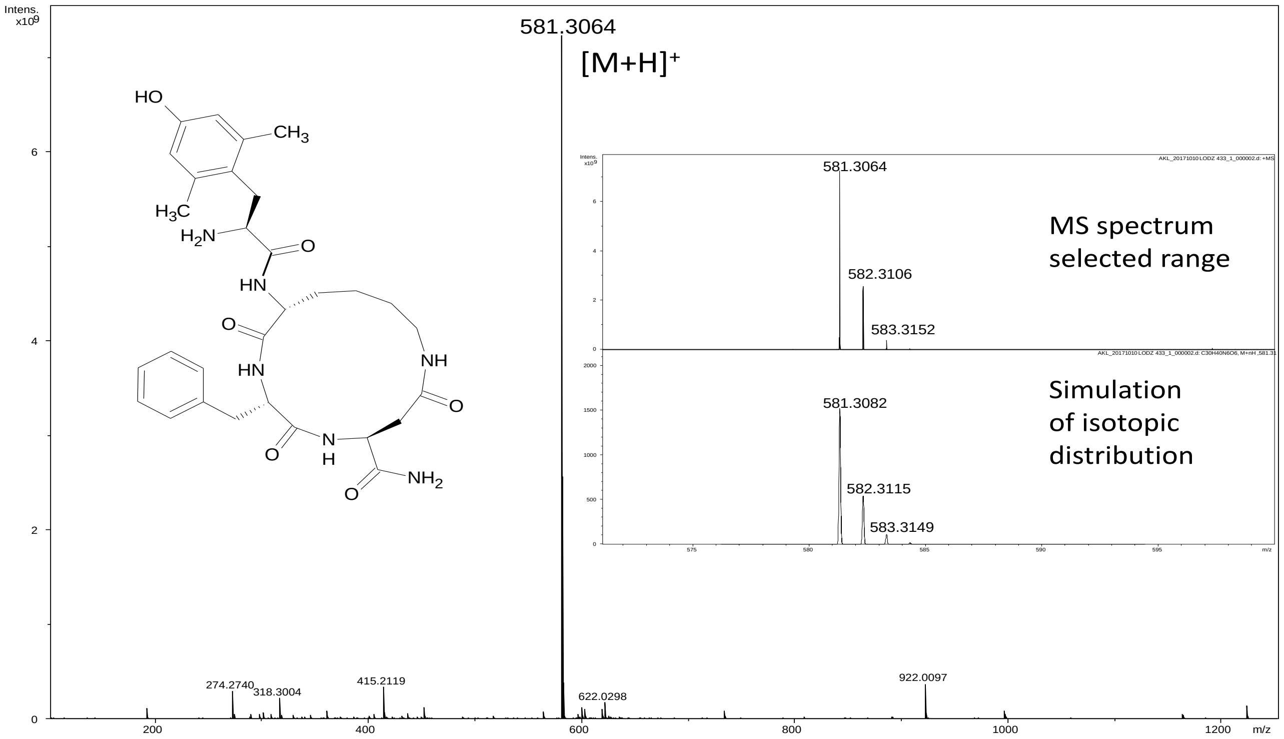

Fig.S3. High resolution MS spectrum of peptide Dmt-c[D-Lys-Phe-Asp]NH_2 (**C-33**). In inset, fragment of the experimental spectrum is compared with the simulated isotopic profile calculated for the expected molecular formula of protonated species [M+H]+ (bottom panel).

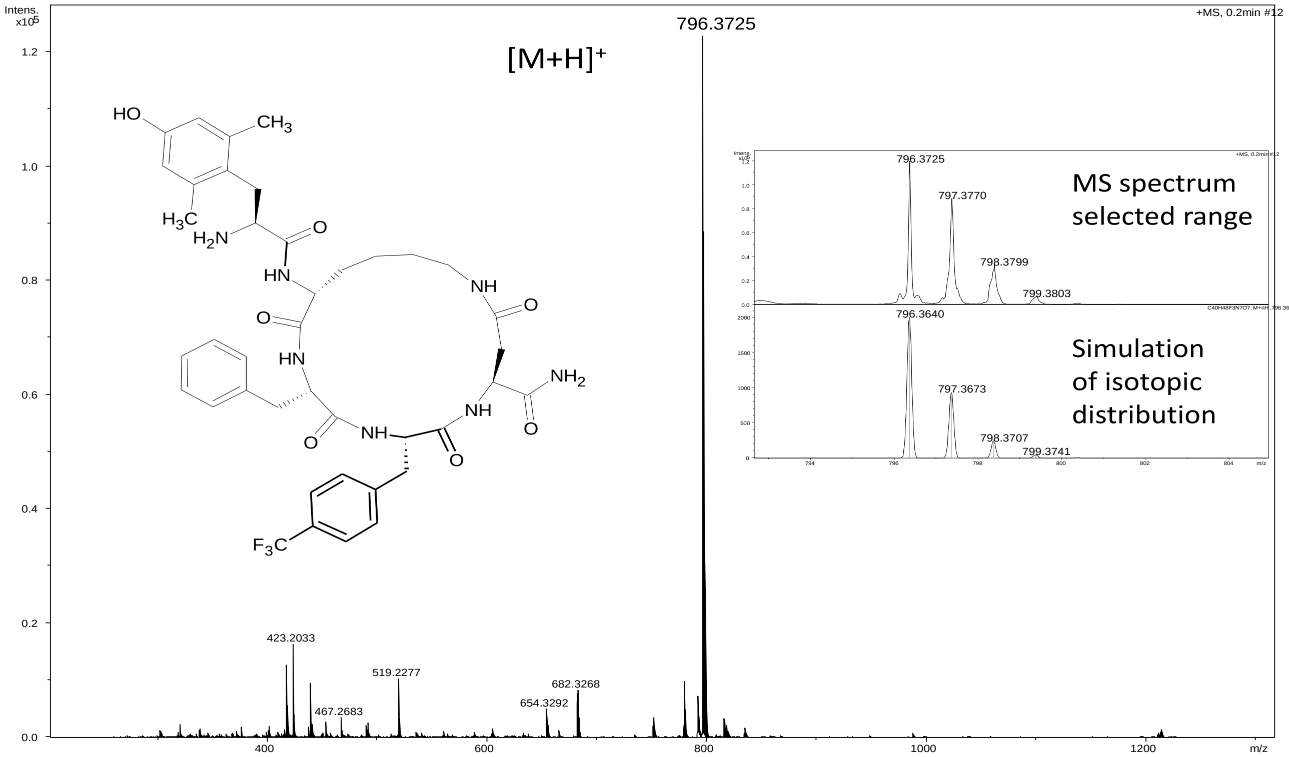

Fig. S4. High resolution MS spectrum of peptide Dmt-c[D-Lys-Phe-pCF_3-Phe-Asp]NH_2 (**F-81**). In inset, fragment of the experimental spectrum is compared with the simulated isotopic profile calculated for the expected molecular formula of protonated species $[M+H]^+$ (bottom panel).

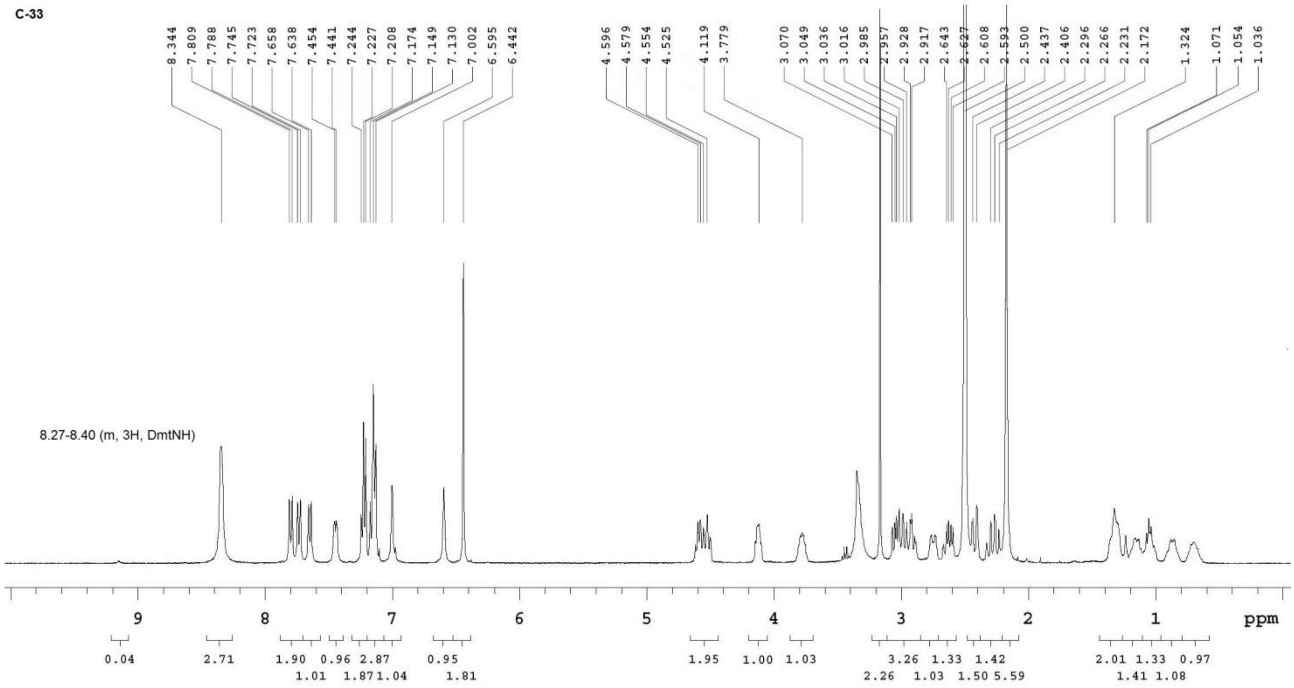

Fig. S5. ^{1}H NMR spectra of **C-33** at 400 MHz in DMSO‑d6.

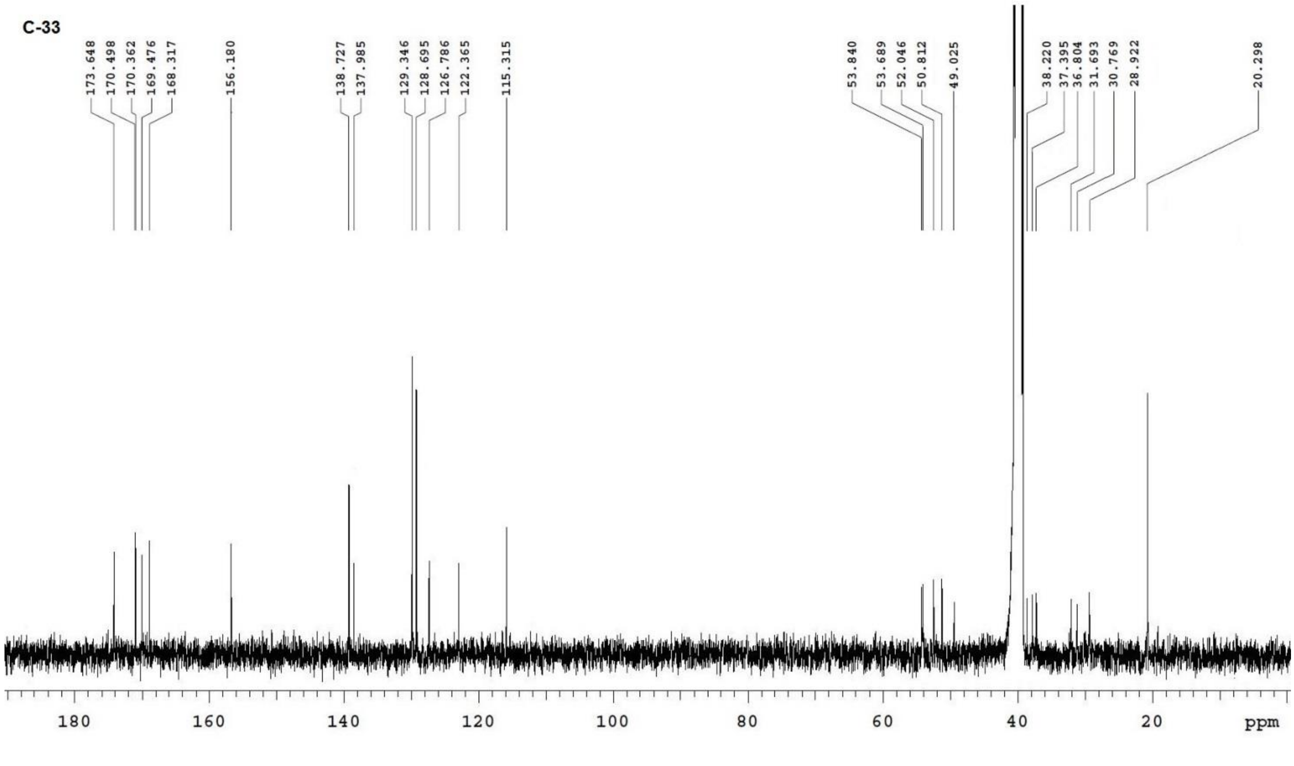

Fig. S6 . ^{13}C NMR spectra of **C-33** at 400 MHz in DMSO‑d6.

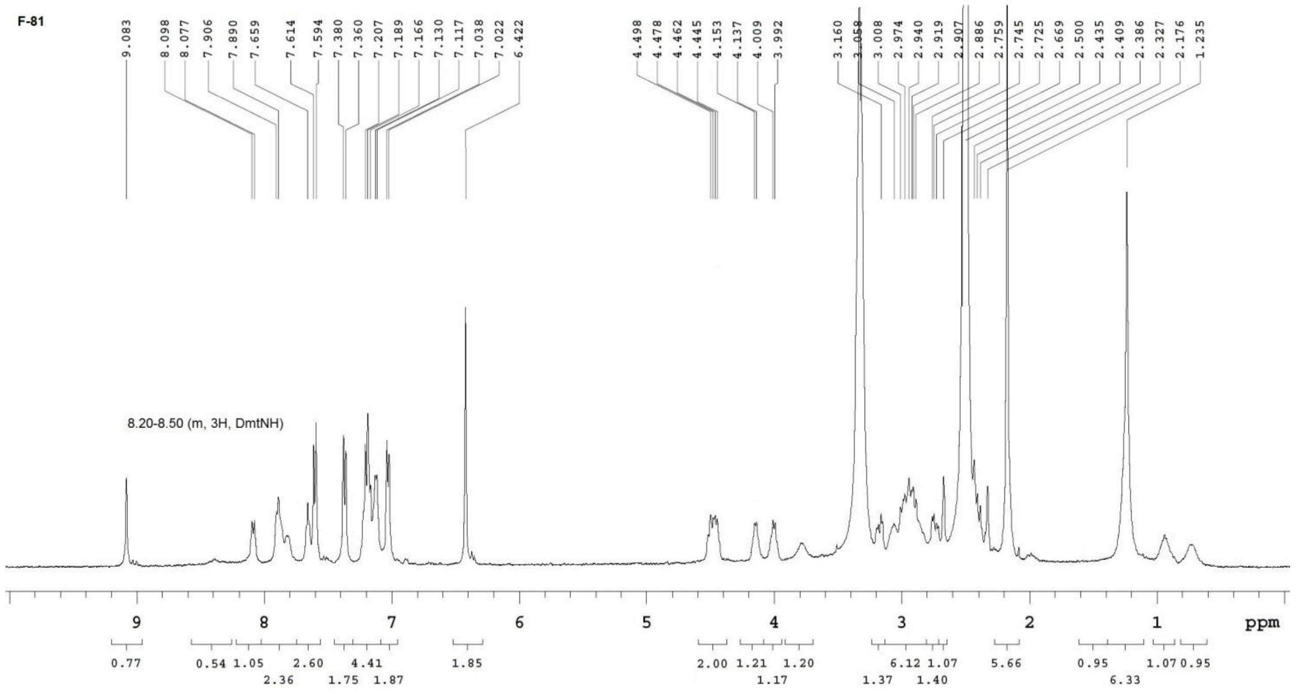

Fig. S7. ^{1}H NMR spectra of **F-81** at 400 MHz in DMSO-d6.

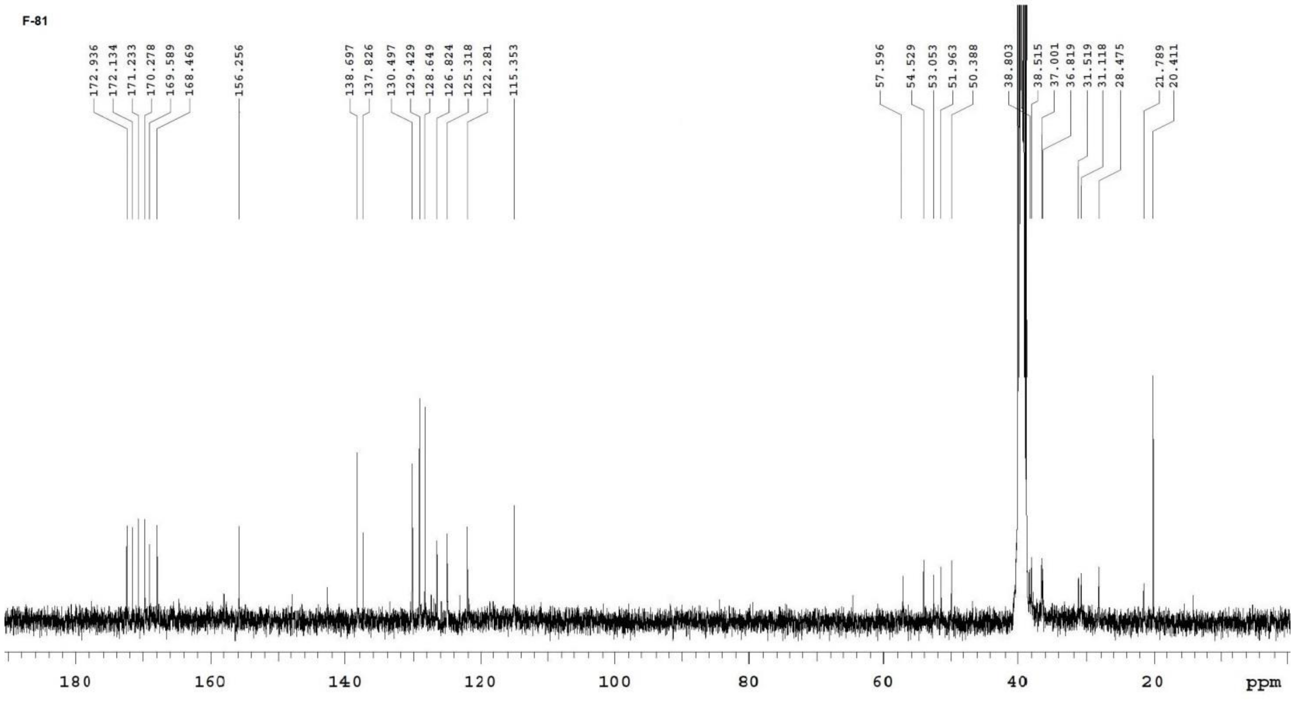

Fig. S8. ^{13}C NMR spectra of **F-81** at 400 MHz in DMSO-d6.

Table S2. Opioid receptor affinity and enzymatic stability of cyclic peptides and EM-2.

compd	Ki[a] [nM]			Enzymatic stability Area [%] 120 min	
	MOP	DOP	KOP	DPP IV	Homogenate
EM-2	2.50±0.05	>1000	>1000	1.9±0.03	6.5±0.15
**F-81**[b]	2.4±0.07	>1000	31.1±2.03	92.1±3.27	90.7±3.14
**C-33**[c]	0.24±0.0[c]	1.33±0.06[c]	79±20[c]	91.1±4.21	89±2.27

[a] Displacement of [^{3}H]DAMGO (MOP-selective), [^{3}H]deltorphin-2 (DOP-selective) and [^{3}H]U-69593 (KOP-selective) from human opioid receptor membrane binding sites. b From ref. [1]. c From ref. [2]. All values are expressed as mean ± SEM, n ≥ 3.

Table S3. Effect of reference agonists and cyclopeptides at human recombinant opioid receptors coupled to calcium signaling *via* chimeric G proteins.

compd	MOP		DOP		KOP	
	pEC$_{50}$ (CL$_{95\%}$)	α±SEM[b]	pEC$_{50}$ (CL$_{95\%}$)	α±SEM	pEC$_{50}$ (CL$_{95\%}$)	α±SEM
EM-2[a]	7.83 (7.69-7.96)	1.00	inactive		inactive	
DPDPE [a]	inactive		7.33 (7.20-7.47)	1.00	inactive	
Dyn A[a]	6.67 (6.17-7.17)	0.83±0.10	7.73 (7.46-8.00)	0.99±0.04	8.81 (8.73-8.90)	1.00
**F-81**[a]	8.19 (7.93-8.44)	0.8±0.05	inactive		8.22 (7.75-8.69)	0.63±0.04
**C-33**[b]	8.53 (8.25-8.81)	1.03±0.06	7.65 (6.74-8.55)	0.98±0.05	6.11 (5.80-6.42)	0.5±0.02

[a] From ref. [1]. [b] From ref. [2]. All values are expressed as mean ± SEM, n ≥ 5.

Table S4. Potencies, maximal effects, and bias factors (calculated by using EM-2 as a standard unbiased ligand) of EM-2 and analogs **F-81** and **C-33**.

compd.	MOP/G-protein		MOP/*β*-arrestin 2		bias factor (CL$_{95\%}$)
	pEC$_{50}$ (CL$_{95\%}$)	α±SEM	pEC$_{50}$ (CL$_{95\%}$)	α±SEM	
EM-2[a]	7.32 (7.14-7.51)	1.00	7.20 (7.08-7.33)	1.00	0.00
**F-81**[a]	8.28 (7.94-8.62)	0.82±0.04	inactive		-
**C-33**[b]	8.23 (8.09-8.36)	1.05±0.03	8.39 (8.33-8.45)	1.43±0.06	-1.16 (-1.59 - -0.74)

[a] From ref. [1]. [b] From ref. [2]. All values are expressed as mean ± SEM, n ≥ 3.

Table 5. ROESY cross peaks for **F-81** in 8:2 [D6]DMSO/H_2O, rt; vs = very strong, s = strong, m = medium, w = weak.

**Cross Peak**	**Intensity**	**Cross Peak**	**Intensity**
DmtNH-DmtMe	m	LysNH-DmtNH	w
DmtNH-DmtHβ	vs	LysNH-DmtMe	m
DmtNH-DmtHα	vs	LysNHε-LysHγ0.7	m
AspNH-LysHβ	w	LysNHε-LysHγ0.9	m
AspNH-AspHβ	vs	LysNHε-LysHδ	s
AspNH-CF_3PheHβ2.9	m	LysNHε-LysHβ	w
AspNH-CF_3PheHβ3.1	w	LysNHε-AspHβ	vs
AspNH-CF_3PheHα	vs	LysNHε-LysHε2.9	s
AspNH-AspHα	m	LysNHε-LysHε3.0	s
PheNH-PheHα	s	LysNHε-AspHα	s
PheNH-LysHα	s	CF_3PheArH-CF_3PheHβ2.9	vs
PheNH-AspNH	w	CF_3PheArH-CF_3PheHβ3.1	s
PheNH-CF_3PheNH	w	CF_3PheArH-PheHβ2.8	w
PheNH-LysHβ	m	CF_3PheArH-CF_3PheHα	s
PheNH-LysHγ0.9	w	PheArH-PheHβ2.7	vs
PheNH-PheHβ2.7	m	PheArH-PheHβ2.9	s
PheNH-LysHε2.9	w	CONH2-AspHβ	m
PheNH-PheHβ2.9	s	CONH2-AspHα	vs
PheNH-DmtMe	w	CONH2-CF_3PheHα	w
CF_3PheNH-LysHγ0.7	w	DmtArH-LysHδ	w
CF_3PheNH-LysHγ0.9	w	AspHα-AspHβ	vs
CF_3PheNH-LysHβ	w	AspHα-LysHε3.0	w
CF_3PheNH-PheHβ2.7	s	CF_3PheHα-CF_3PheHβ2.9	s
CF_3PheNH-CF3PheHβ2.9	s	CF_3PheHα-CF_3PheHβ3.1	s
CF_3PheNH-PheHβ2.9	m	CF_3PheHα-PheHα	w
CF_3PheNH-CF_3PheHβ3.1	w	LysHα-LysHγ0.7	w
CF_3PheNH-PheHα	s	LysHα-LysHγ0.9	m
CF_3PheNH-CF_3PheHα	m	LysHα-LysHδ	s
CF_3PheNH-CF_3PheArH	m	LysHα-LysHβ	vs
LysNH-LysHγ0.7	m	LysHα-DmtHα	w
LysNH-LysHγ0.9	w	PheHα-PheHβ2.7	s
LysNH-LysHβ	s	PheHα-PheHβ2.9	m
LysNH-DmtHβ	m	DmtHα-LysHβ	w
LysNH-DmtHα	vs	DmtHα-DmtHβ	vs
LysNH-LysHα	m	DmtHα-DmtMe	s
LysNH-LysNHε	w	DmtHβ-DmtMe	vs
LysNH-CF_3PheNH	w	DmtMe-LysHβ	m
LysNH-PheNH	m		

Table 6. ROESY cross peaks for **C-33** in 8:2 [D6]DMSO/H_2O, rt; vs = very strong, s = strong, m = medium, w = weak.

Cross Peak	Intensity	Cross Peak	Intensity
DmtNH-DmtMe	s	LysNHε-LysHγ0.9	m
DmtNH-DmtHβ2.9	s	LysNHε-LysHβ/δ1.3	s
DmtNH-DmtHβ3.0	m	LysNHε-AspHβ2.3	s
DmtNH-DmtHα	vs	LysNHε-AspHβ2.4	w
DmtNH-LysHα	w	LysNHε-LysHε2.8	vs
DmtNH-PheArH	m	LysNHε-LysHε3.3	m
DmtNH-LysNH	s	LysNHε-AspHα	w
DmtNH-PheNH	w	LysNHε-$CONH_2$6.6	w
AspNH-LysHγ0.9	s	PheArH-DmtMe	w
AspNH-LysHβ1.3	m	PheArH-PheHα	s
AspNH-AspHβ2.3	m	$CONH_2$7.0-LysHγ0.9	w
AspNH-AspHβ2.4	m	$CONH_2$7.0-AspHβ2.4	w
AspNH-LysHε2.8	w	$CONH_2$7.0-AspHα	m
AspNH-LysHα	w	$CONH_2$6.6-LysHγ0.9	m
AspNH-AspHα	m	$CONH_2$6.6-LysHδ1.3	w
AspNH-PheHα	m	$CONH_2$6.6-AspHβ2.4	w
AspNH-$CONH_2$6.6	s	$CONH_2$6.6-LysHα	w
AspNH-$CONH_2$7.0	m	$CONH_2$6.6-AspHα	m
AspNH-LysNHε	m	PheHα-PheHβ2.6	s
AspNH-PheNH	m	PheHα-PheHβ3.0	s
PheNH-LysHγ0.9	w	PheHα-LysHα	w
PheNH-LysHβ1.0	w	AspHα-AspHβ2.3	s
PheNH-LysHβ1.3	m	AspHα-AspHβ2.4	s
PheNH-PheHβ2.6	s	LysHα-LysHγ0.7	s
PheNH-PheHβ3.0	w	LysHα-LysHγ0.9	w
PheNH-LysHα	w	LysHα-LysHβ1.0	vs
PheNH-PheHα	m	LysHα-LysHβ1.3	w
PheNH-PheArH	s	LysHα-DmtMe	w
PheNH-LysNHε	w	LysHα-DmtHβ3.0	w
PheNH-LysNH	vs	LysHα-DmtHα	w
LysNH-LysHβ1.3	m	DmtHα-DmtMe	vs
LysNH-DmtMe	w	DmtHα-DmtHβ2.9	s
LysNH-PheHβ2.6	w	DmtHα-DmtHβ3.0	m
LysNH-DmtHβ3.0	w	LysHε3.3-LysHδ1.1	s
LysNH-DmtHα	w	LysHε3.3-LysHδ1.3	s
LysNH-LysHα	m	LysHε3.3-AspHβ2.4	w
LysNH-PheHα	w	LysHδ1.3-LysHγ0.7	s
LysNH-DmtArH	w	LysHδ1.3-LysHγ0.9	w
LysNH-PheArH	m		

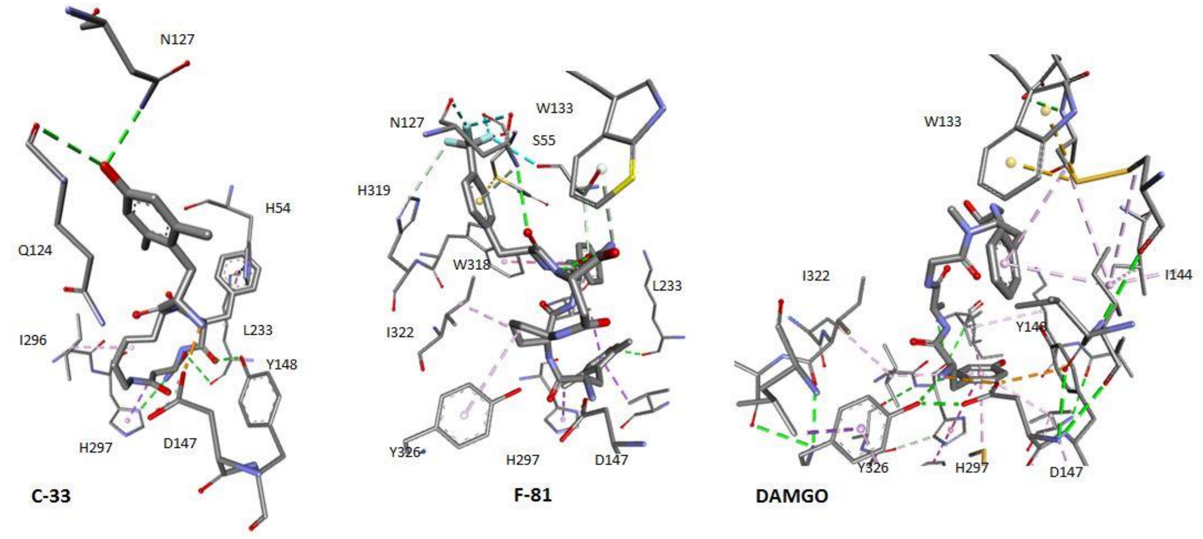

Fig. S9. Rear views of the binding modes for peptides **C-33**, **F-81**, to the deposited structure of MOP (PDB ID: 6DDF); the pose of DAMGO extracted from 6DDF is also shown for comparison. The relevant receptor residue side chains are rendered in thick lines and the ligands in sticks; C is rendered in grey, N in blue, O in red. Dashed green lines represent conventional hydrogen bonds, while cation–π interactions are rendered in yellow, π–π interactions in violet, hydrophobic interactions in white, other interactions in pink.

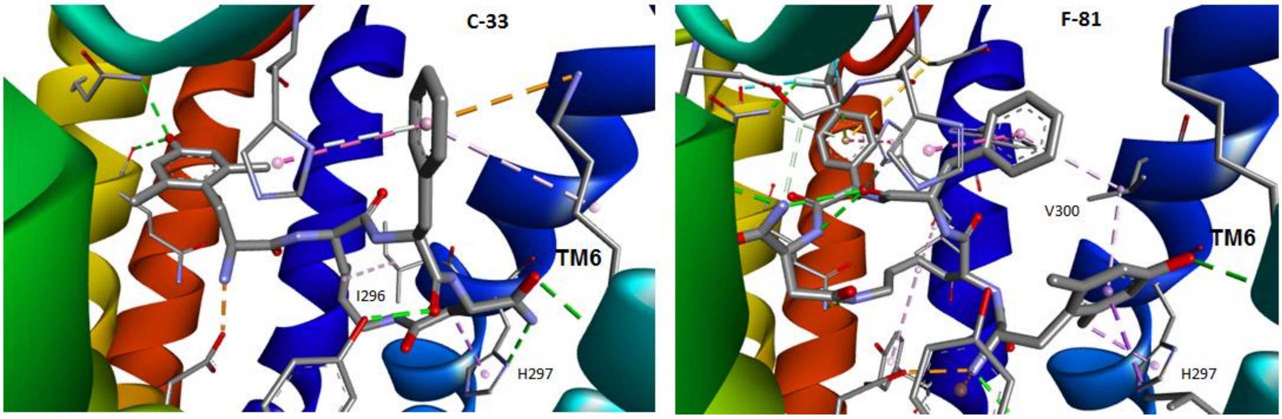

Fig. S10. Details of the interactions between **C-33** or **F-81** and TM6. The ligands are rendered in sticks; C in grey, N in blue, O in red; dashed green lines represent conventional hydrogen bonds, while cation–π interactions are rendered in yellow, π–π interactions in violet, hydrophobic interactions in white, other interactions in pink.

1. Piekielna-Ciesielska, J.; Ferrari, F.; Calo', G.; Janecka, A. Cyclopeptide Dmt-[D-Lys-p-CF3-Phe-Phe-Asp]NH2, a novel G protein-biased agonist of the mu opioid receptor. Peptides 2018, 101, 227-233.

2. Gach-Janczak, K.; Piekielna-Ciesielska, J.; Adamska-Bartłomiejczyk, A.; Wtorek, K.; Ferrari, F.; Calo', G.; Szymaszkiewicz, A.; Piasecka-Zelga, J.; Janecka A. In vitro and in vivo activity of cyclopeptide Dmt-c[D-Lys-Phe-Asp]NH_2, a mu opioid receptor agonist biased toward β-arrestin. *Peptides* **2018**, *105*, 51-57
